# Emerging Biomedical Engineering Therapies for Infected Diabetic Foot Ulcers: Toward Antibacterial Functionalization and Pathology‐Responsive Regulation

**DOI:** 10.1002/smsc.202500482

**Published:** 2025-11-14

**Authors:** Yaqi Yao, Mengyi Huang, Yuetong Li, Yiqi Lin, Junjie Dong, Jianhang Du, Aixia Zhai, Changlong Bi, Luoyuan Li

**Affiliations:** ^1^ Department of Endocrinology and Metabolic Diseases The Key Laboratory of Diabetes Prevention and Treatment in Guangdong Province The Eighth Affiliated Hospital of Sun Yat‐sen University Shenzhen Guangdong 518033 China; ^2^ Biological Laboratory of Hetao Shenzhen‐Hong Kong Science and Technology Innovation Cooperation Zone The Eighth Affiliated Hospital of Sun Yat‐sen University Shenzhen Guangdong 518033 China

**Keywords:** antimicrobial strategies, biomedical engineering therapies, infected diabetic foot ulcer, responsive antimicrobial biomaterials

## Abstract

Infected diabetic foot ulcer (DFU) endangers patients through complex complications, which seriously increase the risk of amputation, prolongation of disability time and mortality, as well as bring a heavy burden to the medical system. This review focuses on the emerging biomedical engineering therapy of DFU and deeply analyzes the multiple pathogenic factors driving these intractable DFU wounds, including impaired angiogenesis, inflammatory disorder, microbial biofilm formation, and impaired immune response. It also synthesizes current clinical treatments and elaborates on their limitations that underscore the need for innovative solutions. The core of the review delves into recent breakthroughs in responsive antimicrobial biomaterials, emphasizing their stimuli‐triggered mechanisms that enable targeted drug release, enhanced bacterial eradication, and tissue regeneration promotion. Furthermore, it explores future trajectories for multifunctional biomaterials, envisioning integrated systems that combine antimicrobial, anti‐inflammatory, and pro‐healing properties to address the complex pathophysiology of infected DFU. By bridging current clinical challenges with biomaterial innovations, it can provide actionable insights for developing patient‐centric therapeutic strategies in biomedical engineering.

## Graphical abstract

Infected diabetic foot ulcer endangers patients with high amputation, disability, mortality risks, and heavy medical burdens. This review analyzes its pathogenic factors, the limitations of current treatments, the breakthroughs of responsive antimicrobial biomaterials, and the challenge of multifunctional biomaterial innovations for further developing patient‐centric therapeutic strategies in biomedical engineering.

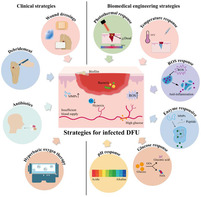

## 1 Introduction

With the global rise in diabetes prevalence, an increasing number of individuals are affected by diabetic foot ulcer (DFU), which is positively correlated with population aging.^[^ ^1^ ^]^ Ulcer formation leads to tissue damage and disruption of the skin barrier, making the wound site highly susceptible to bacterial invasion.^[^ ^2^ ^]^ It is estimated that ≈50%–60% of DFU become infected, and among these patients with infection, 15%–20% ultimately require amputation to control the infection or facilitate healing.^[^ ^3^, ^4^ ^]^ Therefore, infection not only leads to persistent inflammation and tissue necrosis but is also a major contributor to disability and mortality in patients with DFU. The predominant pathogens responsible for infected DFU include *Staphylococcus aureus*, *Escherichia coli*, and *Pseudomonas aeruginosa*, while anaerobic bacteria may be present in the deeper tissue infections.^[^ ^5^, ^6^ ^]^ Typical clinical manifestations include localized pain, swelling, induration, sinus tract formation, purulent discharge, and gangrene. Wounds infected by pathogens are more difficult to heal. Moreover, the microorganisms spread from the surface to the subcutaneous tissue, through tendons to all parts of the foot.^[^ ^7^ ^]^ Besides, it may cause osteomyelitis or disseminate systemically via the bloodstream. Compared to noninfected DFU patients, those with infections have a 55.7‐fold increased risk of hospitalization and a 154.5‐fold increased risk of amputation.^[^ ^8^ ^]^ The five‐year mortality rate for DFU patients is ≈30%, while it exceeds 70% in patients who undergo major amputations.^[^ ^9^ ^]^

Infectious DFUs are a complex pathological process involving multiple factors. To begin with, the development of DFU is associated with both external mechanical factors (e.g., ill‐fitting footwear and excessive plantar pressure) and internal factors (neuropathy, vasculopathy, impaired immune responses).^[^ ^10^, ^11^, ^12^ ^]^ Chronic hyperglycemia contributes to the activation of the polyol pathway and oxidative stress and leads to the accumulation of advanced glycation end‐products (AGEs), which collectively damage microvascular and neural structures, promoting the onset of neuropathy and vasculopathy.^[^ ^13^, ^14^ ^]^ Due to neuropathy‐induced sensory loss, patients often fail to notice minor foot injuries, allowing mechanical stress to exacerbate skin damage and facilitate bacterial invasion. Vascular abnormalities further impair peripheral perfusion and oxygen delivery, thereby hindering neovascularization and delaying wound healing.^[^ ^15^ ^]^ Meanwhile, immune dysfunction and bacterial biofilm formation promote microbial persistence and resistance to treatment, subsequently leading to the progression from superficial ulcer infections to inflammation of deep soft tissues or bones.^[^ ^16^, ^17^ ^]^

Conventional management of infected DFU involves empirical antibiotic therapy followed by targeted antibiotic treatment once the causative pathogens are identified.^[^ ^7^ ^]^ The local use of antibiotics allows drugs to act directly at the site of infection, which can avoid the problem of systemic antibiotics being unable to reach the affected area through the blood vessels, but it remains a subject of controversy.^[^ ^2^, ^18^ ^]^ As a result, antibiotics alone are often insufficient, and adjunctive treatments are required. Surgical debridement to remove necrotic tissues, including infected bone, can clear most of the bacteria.^[^ ^19^ ^]^ In certain cases, skin grafting and revascularization may be considered after infection control to promote healing and prevent amputation. However, current treatment approaches face several major challenges, including the increasing prevalence of antibiotic resistance and poor antibiotic bioavailability. Persistent infections not only impair wound healing but also elevate healthcare costs for patients.^[^ ^20^ ^]^ In recent years, biomaterials have emerged as promising tools in wound care and tissue engineering. These materials offer capabilities such as inflammation modulation, angiogenesis promotion, and extracellular matrix (ECM) remodeling, opening up new ideas for DFU management.^[^ ^21^, ^22^ ^]^ Among them, antimicrobial biomaterials have been developed in various forms, including hydrogels, nanofibers, and microneedles, enabling localized drug delivery, tissue regeneration, and modulation of the wound microenvironment.^[^ ^23^, ^24^, ^25^ ^]^ By integrating various therapeutic mechanisms, antimicrobial biomaterials present a feasible strategy for the comprehensive treatment of infected DFU.

Therefore, this review first introduced the pathogenic factors of infected DFU and current clinical approaches for treatment. Subsequently, we focused on recent advances in responsive antimicrobial biomaterials designed for infected DFU. Finally, we also discussed the future development of multifunctional biomaterials, intending to provide precise, integrated therapeutic and wound management solutions for infected DFU.

## 2 The Pathogenic Factors of Infected DFU

### 2.1 Pathological Changes of DFU

#### 2.1.1 Peripheral Neuropathy

Peripheral neuropathy caused by hyperglycemia includes sensory, motor, and autonomic disorders. The typical symptoms of diabetic peripheral neuropathy often begin in the toes of both feet, manifesting as symmetric pain, numbness, and a reduction or loss of temperature and pain sensation, eventually progressing to a “sock‐like” sensory deficit in the feet and legs.^[^ ^26^ ^]^ Consequently, patients may not be able to easily detect minor injuries in their feet. Besides, peripheral sensorimotor neuropathy causes sensory deficits, muscle weakness, and muscle atrophy. These changes lead to an abnormal gait and an abnormal increase in plantar pressure, which is often closely associated with ulcer formation.^[^ ^27^, ^28^ ^]^ Studies indicate that motor peripheral neuropathy can cause changes in foot morphology, such as second toe deformities, bowed feet, and pronated feet, thus increasing the risk of DFUs.^[^ ^29^ ^]^ More critically, autonomic neuropathy leads to decreased sweat secretion.^[^ ^30^ ^]^ Reduced sweating results in dry, cracked skin, increasing its vulnerability and impairing its ability to repair, which is also a risk factor for DFU.^[^ ^31^, ^32^ ^]^ These wounds or fissures become portals for the infection to invade.^[^ ^33^ ^]^ In addition, peripheral neuropathy could weaken the local defense functions and prolong recovery from foot infections.

#### 2.1.2 Vasculopathy

Hyperglycemia is known to affect vascular homeostasis and ultimately endothelial dysfunction through a variety of mechanisms.^[^ ^34^, ^35^ ^]^ Various vasculopathies, including atherosclerosis, coagulation, and thrombosis, often result in peripheral arterial disease and microvascular dysfunction in DFU patients.^[^ ^36^ ^]^ Vascular disease and impaired peripheral circulation can lead to reduced blood supply to the foot, inadequate nutrient supply, and ultimately cause localized ulceration or even complete foot necrosis.^[^ ^37^ ^]^ Insufficient blood supply simultaneously reduces the local anti‐infection ability, weakens the migration and aggregation ability of immune cells to the infected site, and then reduces the local tissue oxygen partial pressure, inhibits fibroblast proliferation and collagen synthesis, and delays wound healing. In addition, inhibiting the deformability of red blood cells may hinder capillary blood flow in DFU patients, and the combined effect with vascular stenosis further impedes blood flow to the site of injury, and then leading to poor wound healing.^[^ ^38^ ^]^ In addition to these factors, vascular regenerative maturation, one of the key factors in wound healing, is similarly inhibited in diabetes.^[^ ^39^ ^]^ Immune cells and antimicrobial drugs do not reach the wound through the bloodstream, therefore limiting bacterial clearance. Meanwhile, hyperglycemia can also inhibit the chemotaxis, phagocytosis, and bactericidal functions of neutrophils, as well as affect the metabolic pathway of glucose in cells, weakening their bactericidal effect. Long‐term hyperglycemia leads to nonenzymatic glycation of proteins, affecting tissue repair ability and antibody function, and further increasing susceptibility to infection.

#### 2.1.3 Immune Dysregulation

The immune dysfunction of DFU patients makes them more susceptible to foot infections.^[^ ^40^, ^41^ ^]^ In the hyperglycemic environment, immune dysfunction weakens the ability of macrophages to polarize toward an anti‐inflammatory (M2) type, while the dominant proinflammatory (M1) type polarization leads to uncontrolled local inflammatory responses.^[^ ^42^ ^]^ This excessive inflammation damages surrounding healthy tissues, hindering repair and infection control. A hyperglycemic environment could inhibit the expression of surface adhesion molecules (CD11b/CD18) in neutrophils, weakening their adhesion ability to endothelial cells, for inhibiting their migration to the site of foot infection. The migration phenotype of immune cells in diabetic skin is impaired, characterized by the inhibition of interleukin‐13 (IL‐13) and IFN‐γ, along with dysregulation of biological processes such as monocyte migration, dendritic cell migration, and chemotaxis of antigen‐presenting cells, which is a mechanism behind unhealing DFU.^[^ ^43^ ^]^ Besides, compared to normal tissue, DFU tissue contains a higher proportion of resting NK cells, M0 macrophages, and activated mast cells, whereas activated resting mast cells and NK cells are relatively reduced.^[^ ^44^ ^]^ The imbalance in the ratio of these immune cells is detrimental to the resistance and clearance of DFUs from various microbial infections.^[^ ^45^, ^46^ ^]^

### 2.2 Pathogen Characteristics of Infected DFU

Bacterial infections are common in diabetic patients, especially foot ulcers that provide an entry point for pathogens to invade.^[^ ^47^ ^]^ The hyperglycemic and hypoxic wound microenvironment provides favorable conditions for bacterial colonization and proliferation.^[^ ^48^ ^]^ Once microorganisms colonize the wound, they can further spread to subcutaneous tissues, including fascia, tendons, muscles, joints, and bones.^[^ ^7^ ^]^ In addition to causing osteomyelitis, it may also cause severe systemic symptoms. Infected foot ulcer is a key factor leading to amputation in patients with DFU.^[^ ^4^ ^]^

#### 2.2.1 Category of Pathogens

The most common bacterial species isolated from DFU include *P. aeruginosa*, *S. aureus*, and *E. coli*.^[^ ^49^ ^]^ Among these, *S. aureus* is the most frequently isolated pathogen.^[^ ^50^ ^]^ *S. aureus* produces a variety of virulence factors (VFs) such as coagulases, proteases, hemolysins, and collagenases, which enhance bacterial adhesion at the wound site and create a favorable environment for colonization and tissue invasion.^[^ ^51^ ^]^ The production of these VFs is predominantly fueled by glucose. Under hyperglycemic conditions, *S. aureus* upregulates its glycolytic activity by acquiring high‐affinity glucose transporters, GlcA and GlcC, which in turn enhance its pathogenic potential.^[^ ^52^ ^]^ Some strains also produce staphyloxanthin, which enhances resistance to oxidative stress, increases bacterial survival in neutrophils, and significantly delays wound healing.^[^ ^53^ ^]^

*E. coli*, a Gram‐negative opportunistic pathogen, is also commonly found in DFUs. It harbors VF genes encoding toxins such as P fimbriae, S fimbriae, Siderophore, hemolysin, and cytotoxic‐necrotizing factors.^[^ ^54^ ^]^ These virulence determinants enable *E. coli* to adhere to host cells, survive within the host environment, and damage tissues, particularly in immunocompromised individuals.^[^ ^55^ ^]^ In addition, the lipopolysaccharide (LPS) component of the Gram‐negative outer membrane can activate the LPS‐TLR4 signaling pathway, triggering inflammatory cell infiltration and contributing to a sustained inflammatory state in DFU.^[^ ^56^ ^]^

*P. aeruginosa* is another Gram‐negative bacterium frequently associated with chronic wound infections. Its lectin protein, LecB, binds and cross‐links fucosylated receptors on the apical surface of epithelial cells, initiating signal transduction cascades via Src kinases and phosphoinositide 3‐kinase (PI3K), thereby facilitating bacterial invasion through the apical membrane.^[^ ^57^ ^]^ Furthermore, LecB also enables *P. aeruginosa* to transition between planktonic, intracellular, and biofilm states, allowing it to evade immune responses and resist antibiotic treatments.^[^ ^58^ ^]^

Notably, DFUs are typically colonized by complex polymicrobial communities rather than a single pathogen. The synergistic and competitive interactions among various bacterial species contribute to chronic inflammation, impaired healing, and resistance in DFUs.

#### 2.2.2 Formation and Drug Resistance of Bacterial Biofilm

**Figure 1:**
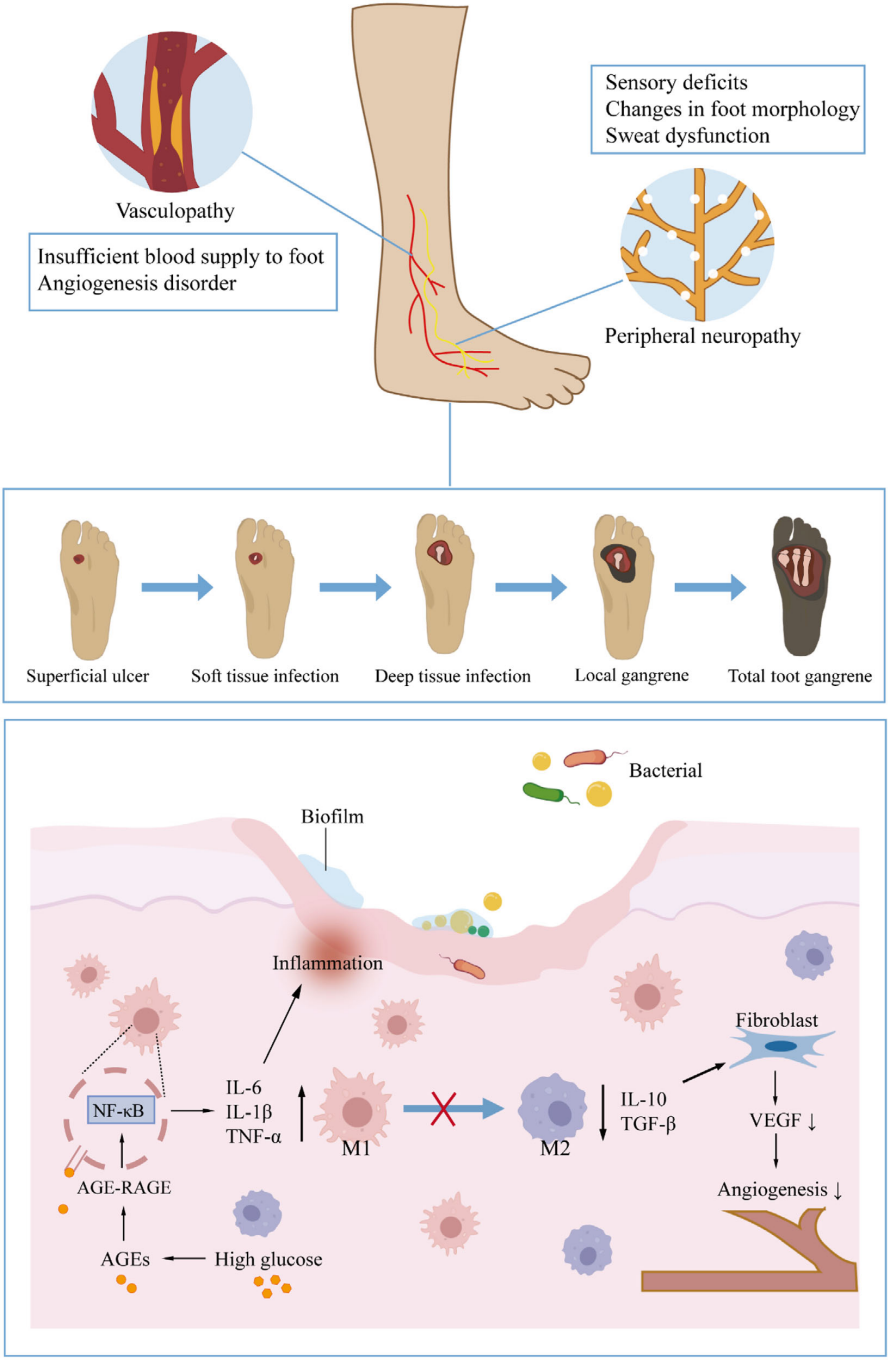
The pathogenic factors of infected DFU. AGEs, advanced glycation end‐products; RAGE, receptor for advanced glycation end‐products; NF‐κB, nuclear factor kappa‐B; M1, classically activated macrophage; M2, alternatively activated macrophage; IL, interleukin; TNF‐α, tumor necrosis factor‐α; TGF‐β, transforming growth factor‐β; VEGF, vascular endothelial growth factor.

Most diabetic foot infections involve polymicrobial colonization, including aerobic and anaerobic species, which form biofilms on the wound surface.^[^ ^59^ ^]^ Bacteria are enveloped by extracellular polysaccharide matrix, which increases drug resistance enormously compared to free bacteria.^[^ ^60^, ^61^ ^]^ This matrix, composed of polysaccharides, proteins, lipids, nucleic acids, and other biomolecules, plays crucial structural and functional roles in biofilm formation and maintenance.^[^ ^62^ ^]^ Extracellular polysaccharide matrix facilitates microbial adhesion to both biotic and abiotic surfaces, maintains proximity among cells, and enables intercellular communication within confined microenvironments.^[^ ^63^ ^]^ Moreover, the presence of extracellular polymer matrix is seen as a physical barrier that bacteria are able to evade recognition by host immune cells and resistance to antimicrobial therapies, leading to long‐term chronic and recurrent infections.^[^ ^64^ ^]^ Biofilms are dynamic structures that continuously remodel in response to local microenvironmental changes. Through quorum sensing (QS), which is a cell‐to‐cell communication mechanism mediated by chemical signaling, bacteria can regulate gene expression and metabolic activity in a coordinated manner, further promoting the pathogenicity of biofilm.^[^ ^65^ ^]^ Notably, high levels of oxidative stress in DFU reduce the diversity of the bacterial wound microbiome and promote bacterial colonization of the skin microbiome to form biofilms (**Figure** **1**).^[^ ^66^ ^]^

## 3 Treatment Strategies for Infected DFU

### 3.1 Antimicrobial Drugs

Infected ulcers almost always require antimicrobial therapy, which can be administered systemically (by oral, intravenous, or intramuscular administration throughout the body) or topically (by applying antimicrobial preparations in the form of solutions, gels, and ointments).^[^ ^67^ ^]^ Mild infections are typically treated with first‐generation cephalosporins, macrolides, or fluoroquinolones, while moderate to severe infections usually require second‐/third‐generation cephalosporins, β‐lactams, or combination antibiotic therapy.^[^ ^33^ ^]^ Some common antibiotics are summarized in **Table** **1**. Appropriate antimicrobial drugs are usually selected based on the results of clinical bacterial cultures.

**Table 1:** Common antibiotics for DFU.

Type	Bactericidal mechanism	Drug resistance mechanisms	representative	Antimicrobial spectrum	Advantages	Disadvantages	Ref.
β‐lactam	Inhibition of bacterial cell wall synthesis	1) Production of β‐lactamase 2) Changing the structure of PBPs 3) Enhanced drug efflux 4) Lack of autolytic enzymes	Amoxicillin/clavulanate	Most G^+^ bacteria, G^−^ bacilli, and anaerobes	Relief of diabetic foot osteomyelitis	Hepatotoxicity and gastrointestinal reactions	[223]
			Piperacillin/tazobactam	Broad spectrum, including *Pseudomonas aeruginosa*	Treatment for moderate to severe DFI	Renal toxicity, liver toxicity, leukopenia, and thrombocytopenia	[224]
			Ertapenem	Most of the pathogens, including MRSA, streptococci, Enterobacteriaceae, and anaerobes	Effective for moderate to severe DFI	Invalid for *Enterococcus* or *Pseudomonas* spp.	[225]
			Ceftobiprole	Broad spectrum	High eradication rate of MRSA	Limited activity against anaerobic bacteria such as *Bacteroides fragilis* and non‐fragilis *Bacteroides* spp.	[226]
Polypeptide antibiotics	Inhibition of bacterial cell wall synthesis	Enzymes that produce the peptidoglycan precursor of the cell wall	Vancomycin	G^+^ bacteria	Frontline drugs for MRSA	Nephrotoxicity	[227]
Aminoglycosides	1) Inhibition of protein synthesis 2) Destroy the integrity of the cell membrane	1) Reduced bacterial membrane permeability. 2) An amino acid on the bacterial ribosome 30S subunit target protein is replaced. 3) The production of a modifying enzyme renders the drug inactive.	Gentamicin	Various G^+^ and G^−^ bacteria, including *Pseudomonas aeruginosa*	Effective for bone and soft tissue infections	High risk of ototoxicity and nephrotoxicity	[228]
Quinolones	Affecting nucleic acid metabolism	1) The production of a modifying enzyme renders the drug inactive. 2) Gene mutations reduce the affinity of enzymes for drugs.	Mupirocin	G^+^ bacteria, including MRSA	Stimulating growth factor production and proliferation of human keratinocytes	Increased usage leads to drug resistance	[229, 230]
			Ciprofloxacin	Various G^+^ and G^−^ bacteria	Effective against some strains resistant to antibiotics such as penicillin.	May induce tendonitis	[231, 232]

#### 3.1.1 β‐Lactam Antibiotics

β‐Lactam antibiotics, including penicillins, cephalosporins, and β‐lactamase inhibitors, are a broad class of antimicrobial agents characterized by the presence of a β‐lactam ring in their chemical structure. Their primary mechanism of action involves mimicking the d‐alanyl–d‐alanine (d‐Ala–d‐Ala) moiety in peptidoglycan precursors, thereby competitively binding to penicillin‐binding proteins (PBPs) in bacterial cells.^[^ ^68^ ^]^ This interaction inhibits the cross‐linking of peptidoglycan strands, ultimately disrupting bacterial cell wall synthesis and compromising structural integrity. Additionally, β‐lactams can activate endogenous autolysins within bacteria, leading to cell lysis.^[^ ^69^ ^]^ β‐lactamase inhibitors (e.g., clavulanic acid, tazobactam) themselves exhibit weak antimicrobial activity, but they irreversibly bind to β‐lactamases, enzymes that degrade β‐lactam antibiotics, thereby protecting the core antibiotic from enzymatic hydrolysis and restoring its antibacterial efficacy.^[^ ^70^ ^]^ Thus, the combination of β‐lactam antibiotics with β‐lactamase inhibitors broadens the antibacterial spectrum and enhances therapeutic efficacy, especially against some resistant strains. Due to their potent antimicrobial activity and broad‐spectrum efficacy, β‐lactam antibiotics remain one of the most widely used classes of antibiotics in clinical practice.

#### 3.1.2 Polypeptide Antibiotics

Polymyxins are a class of polypeptide antibiotics with a narrow antibacterial spectrum, exhibiting potent bactericidal activity primarily against Gram‐negative (G^−^) bacteria. These agents possess multiple cationic polar groups that interact electrostatically with the negatively charged outer membrane of bacteria. Upon binding to membrane phospholipids, polymyxins disrupt membrane integrity by altering its permeability, leading to leakage of intracellular contents and eventual bacterial death.^[^ ^71^ ^]^ Studies have revealed that polymyxins induce the crystallization of lipopolysaccharides in the outer membrane of Gram‐negative bacteria into hexagonal arrays. This structural rearrangement reduces membrane thickness, increases rigidity and permeability, and ultimately causes membrane disintegration.^[^ ^72^ ^]^ Another polypeptide antibiotic, vancomycin, targets Gram‐positive (G^+^) bacteria and is particularly effective against methicillin‐resistant *S. aureus* (MRSA).^[^ ^73^ ^]^ Its mechanism of action is similar to that of β‐lactams; vancomycin binds to the d‐Ala–d‐Ala termini of peptidoglycan precursors, thereby inhibiting the activity of transglycosylase and transpeptidase enzymes. This blockade prevents peptidoglycan cross‐linking, compromising cell wall integrity and resulting in osmotic lysis of the bacterial cell.^[^ ^74^ ^]^

#### 3.1.3 Aminoglycosides

Aminoglycoside antibiotics, such as gentamicin, exhibit potent bactericidal activity against a broad range of aerobic G^−^ bacilli, including *E. coli*, *P. aeruginosa*, and Proteus species. Their primary mechanism of action involves interference with bacterial protein synthesis. Specifically, aminoglycosides bind with high affinity to the A‐site of the 16S rRNA within the 30S ribosomal subunit, leading to misreading of mRNA and inhibition of accurate protein translation.^[^ ^75^ ^]^ In addition to impairing protein synthesis, aminoglycosides can also adsorb onto the bacterial cell membrane, disrupting its integrity and causing leakage of essential intracellular components.^[^ ^76^ ^]^ Aberrant proteins generated by translation errors are inserted into the cytoplasmic membrane, further compromising membrane function and facilitating the uptake of additional aminoglycoside molecules.^[^ ^75^, ^77^ ^]^ These synergistic mechanisms contribute to the rapid bactericidal effects of aminoglycosides.

#### 3.1.4 Quinolones

Moxifloxacin, a representative fourth‐generation quinolone, exhibits broad‐spectrum bactericidal activity and is considered an ideal therapeutic agent for wound infections.^[^ ^78^ ^]^ Fluoroquinolones exert their antibacterial effects by dual‐targeting bacterial DNA gyrase and topoisomerase IV. They bind to specific domains and conformations of the enzymes, thereby disrupting the DNA replication process and inducing double‐stranded DNA breaks, which underlie their potent bactericidal mechanism.^[^ ^79^ ^]^

### 3.2 Physical Therapy

#### 3.2.1 Debridement

Debridement is regarded as a key element of traditional wound care, which includes both nonmechanical (autolytic, enzymatic) and mechanical methods (sharp/surgical, wet to dry debridement, aqueous high‐pressure lavage, ultrasound, and biosurgery/maggot debridement therapy).^[^ ^80^ ^]^ Currently, the commonly used method is surgical debridement, which can remove hyperkeratotic epidermis and necrotic wound tissue from the wound bed.^[^ ^81^ ^]^ Moreover, surgical debridement reduces bacterial diversity in the wound and has been reported to be more effective than antibiotic treatment.^[^ ^82^ ^]^ However, surgical debridement may also damage healthy granulation tissue, and the procedure can be painful for patients with long treatment times.^[^ ^83^ ^]^ It does not guarantee the healing of DFU wounds, and some patients may still require alternative treatments or even face recurrence or amputation.^[^ ^84^ ^]^ One in four patients will have a recurrence of a persistent infection 10–20 days after debridement.^[^ ^4^ ^]^

#### 3.2.2 Hyperbaric Oxygen Therapy (HBOT)

Hyperbaric oxygen therapy (HBOT) involves completely enclosing the patient in a pressure chamber and having them breathe 100% oxygen at a pressure exceeding 1 atmosphere.^[^ ^85^ ^]^ It raises tissue oxygen tension to the optimal level required for wound healing and enhances the bactericidal activity of white blood cells, particularly against certain anaerobic bacteria.^[^ ^86^ ^]^ Furthermore, HBOT promotes fibroblast proliferation and angiogenesis by activating the HIF‐1α signaling pathway, thus supporting DFU wound healing.^[^ ^87^ ^]^ Numerous studies have shown that HBOT improves the healing rate of DFUs, reduces healing time, and lowers the incidence of major amputations.^[^ ^88^ ^]^ However, it has no significant effect on minor amputations or mortality reduction.^[^ ^89^ ^]^

#### 3.2.3 Skin Substitutes

**Figure 2:**
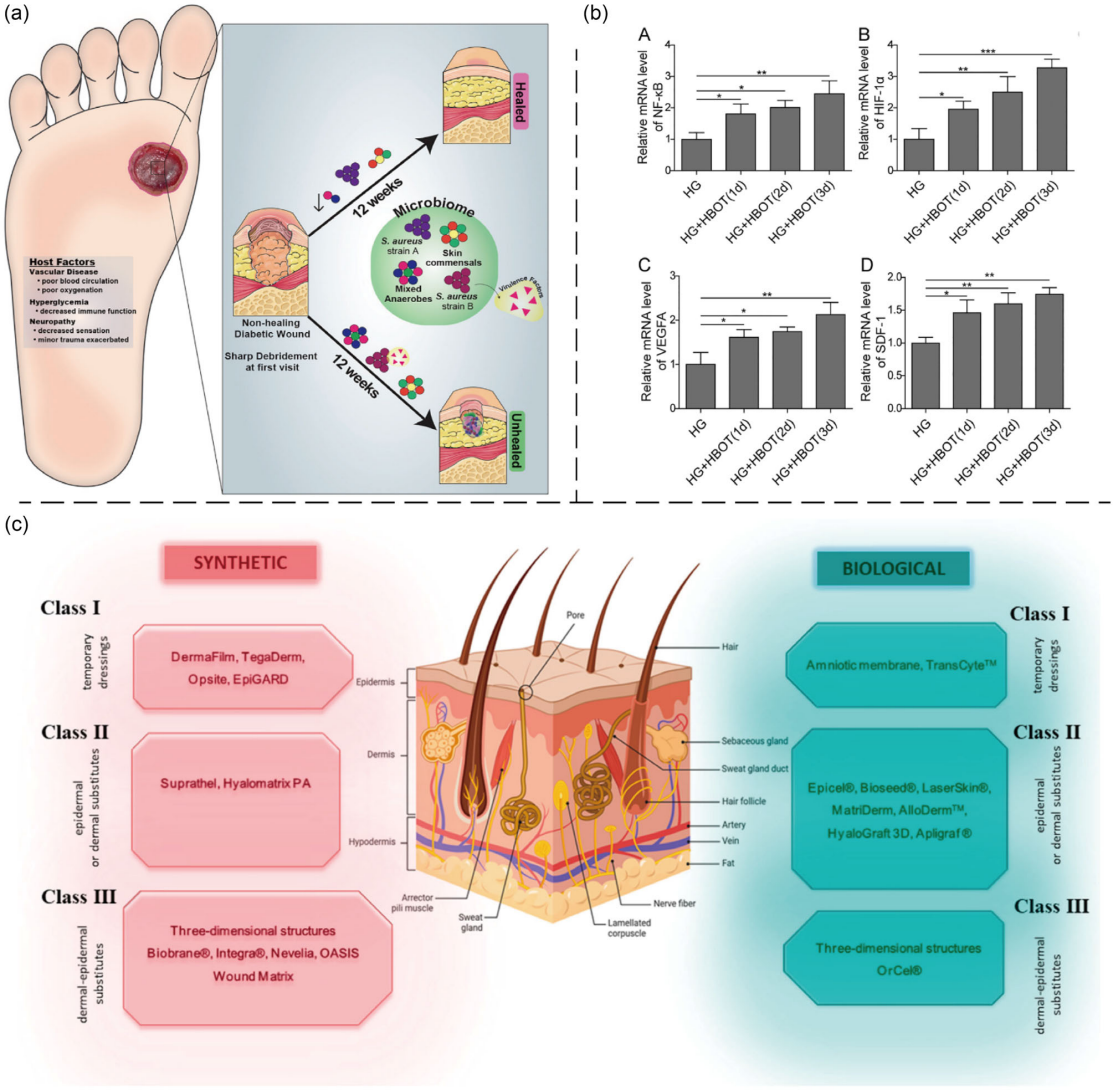
Physical therapy for DFU. a) Schematic diagram of sharp debridement treatment for bacteria. Reproduced with permission.^[^ ^82^ ^]^ Copyright 2019, Elsevier. b) Hyperbaric oxygen activated NF‐κB/HIF‐1α/VEGF/SDF‐1 pathway in human skin fibroblast. Reproduced with permission.^[^ ^87^ ^]^ Copyright 2020, Elsevier. c) Classification of skin substitutes depending on structure and type of biomaterial used. Reproduced with permission.^[^ ^91^ ^]^ Copyright 2024, MDPI.

Artificial skin grafting is a common clinical treatment used for DFU (**Figure** **2**). Skin substitutes mimic the tissue structure and function of natural skin to support wound healing.^[^ ^90^ ^]^ They can close wounds and prevent secondary mechanical damage and bacterial colonization; in addition, they can be used to protect areas where skin grafts have been removed until the skin is epithelialized.^[^ ^91^ ^]^ Skin grafting can enhance the healing rate of DFU and reduce the risk of recurrence.^[^ ^92^, ^93^ ^]^ However, it is generally recommended to perform grafting procedures only after effective infection control. Moreover, some composite skin substitutes involve high manufacturing costs, which may increase the overall financial burden of treatment.^[^ ^94^ ^]^

### 3.3 Medical Device‐Assisted Therapies

Infectious DFUs are frequently accompanied by severe stenosis or occlusion of lower extremity arteries, resulting in local ischemia, which is a major factor contributing to delayed wound healing and worsened infections. Therefore, for ischemic DFUs, promoting lower limb vascular reconstruction and foot angiogenesis is essential for preventing limb amputation.^[^ ^95^ ^]^ The primary goal is to restore pulsatile linear blood flow to the ankle or foot, thereby reestablishing blood supply to the wound site to facilitate healing.^[^ ^96^ ^]^ Within one year following revascularization, the ulcer healing rate ranges from 46% to 91%, demonstrating improvement compared to patients who do not undergo revascularization.^[^ ^97^ ^]^ However, revascularization does not always result in wound healing and may require multiple surgical interventions.^[^ ^98^, ^99^ ^]^

#### 3.3.1 Balloon Angioplasty and Stent Implantation

Percutaneous transluminal balloon angioplasty (PTA) is a minimally invasive endovascular procedure used to dilate narrowed or occluded arteries and veins. A catheter delivers the balloon to the stenotic segment, where inflation temporarily expands the lumen. A stent may be implanted simultaneously to ensure sustained vessel patency. This intervention can effectively promote lower limb revascularization and increase limb salvage rates in diabetic foot patients.^[^ ^100^ ^]^ However, limitations include restenosis due to neointimal hyperplasia post‐procedure, often necessitating repeated interventions. Besides, although stent placement reduces the incidence of restenosis, it still faces challenges related to calcification and Poor adaptation to complex lesions.^[^ ^101^ ^]^ Drug‐coated balloons (DCBs) combine mechanical dilation with local delivery of antiproliferative agents, offering prolonged vessel patency without the need for permanent implants, with an increasing employment in peripheral artery disease (PAD), particularly in femoropopliteal lesions.^[^ ^102^, ^103^ ^]^ However, uncontrolled drug release and high drug loading may lead to distal embolization and increased mortality risk.

#### 3.3.2 Excimer Laser Angioplasty

Excimer laser angioplasty (ELA) employs ultraviolet light at a wavelength of 308 nm, using xenon and hydrogen chloride as active media. The laser delivers pulsed energy through an optical fiber to the lesion site, ablating atherosclerotic plaques and thrombi by breaking molecular bonds.^[^ ^104^ ^]^ Unlike thermal ablation, ELA generates minimal heat, thereby preserving the integrity of vascular endothelium and adjacent tissues. The “cold laser” mechanism and precise photochemical vaporization enable high controllability, targeted ablation, and a reduced incidence of complications such as distal embolization and vascular perforation.^[^ ^105^ ^]^ ELA is often used as an adjunctive therapy to balloon angioplasty and provides another effective option for treating resistant or non‐dilatable lesions.^[^ ^106^ ^]^ Notably, ELA combined with DCB angioplasty has demonstrated superior efficacy in ischemic DFU treatment compared to DCB alone, with an ulcer healing rate of 88.6% and a target vessel patency rate of 80.0%.^[^ ^107^ ^]^

### 3.4 Wound Dressings

Wound dressings are critical in the management of DFUs as they provide a necessary barrier between the ulcer and the external environment. Ideal wound dressings should have the ability to absorb exudate, maintain a moist wound healing environment, avoid wound trauma from removal of the dressing, and protect the wound from microorganisms.^[^ ^108^, ^109^ ^]^ Although a wide variety of dressings are currently available, no single dressing can meet all the requirements, necessitating selection based on the patients’ specific condition.^[^ ^110^ ^]^

#### 3.4.1 Hydrogel

**Figure 3:**
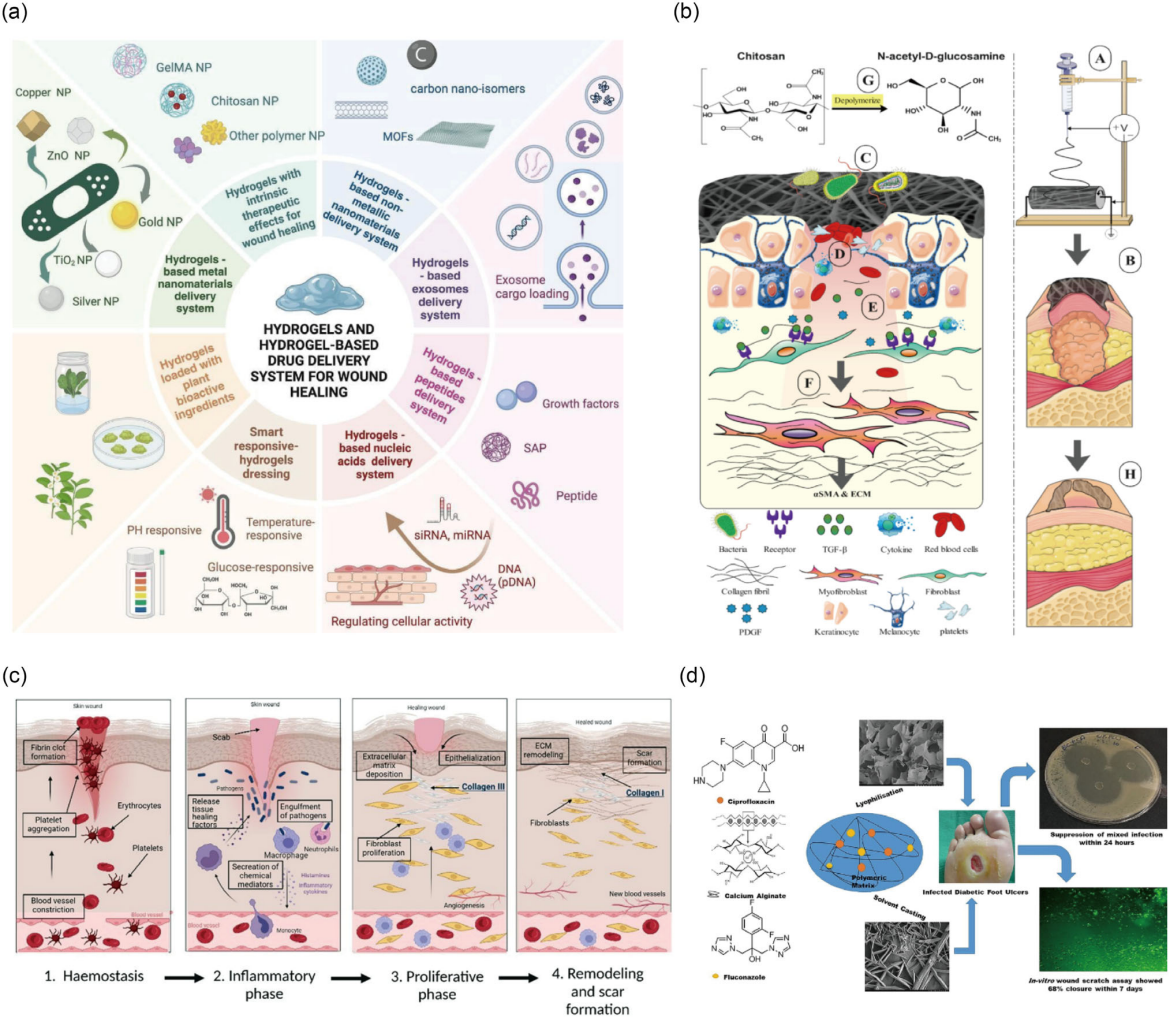
a) Different hydrogel dressings for promoting wound healing. Reproduced with permission.^[^ ^113^ ^]^ Copyright 2025, Elsevier. b) An overview of the role of CS‐based NFs for the healing of DFU. Reproduced with permission.^[^ ^122^ ^]^ Copyright 2023, Elsevier. c) The role of collagen in wound healing. Reproduced with permission.^[^ ^222^ ^]^ Copyright 2022, Elsevier. d) Alginate‐based dressings (films and wafers) for infected DFU. Reproduced with permission.^[^ ^124^ ^]^ Copyright 2021, Elsevier.

Hydrogels, as a novel type of dressing, are considered promising for the treatment of DFUs (**Figure** **3**).^[^ ^111^ ^]^ Compared to traditional dressings, hydrogel dressings enhance granulation tissue formation and epithelialization, reduce the incidence of bacterial infections, and improve the healing rate and shorten the healing time of DFUs.^[^ ^112^ ^]^ Besides, hydrogel has good biocompatibility, bioabsorbability, mechanical stability, and moisturizing properties, and its ability to mimic the dynamic properties of the natural ECM can serve as an ideal wound dressing.^[^ ^113^ ^]^ In addition, loading various bioactive agents or drugs into the dressing, such as growth factors and antibiotics, can better accelerate the healing of DFUs.^[^ ^114^, ^115^ ^]^

#### 3.4.2 Collagen Dressings

Collagen, a major component of native human skin, is biodegradable and biomimetic, making it an ideal material for wound dressing fabrication. Collagen dressings form higher‐order three‐dimensional structures at the cellular level through networking and interaction and can reduce scar size by regulating collagenase activity and ECM degradation through keratinocyte differentiation.^[^ ^116^ ^]^ Compared with conventional foam dressings, collagen dressings have demonstrated superior healing efficacy in DFUs, with complete wound closure rates reaching 82.4%.^[^ ^117^ ^]^ However, native collagen lacks intrinsic antibacterial properties. Therefore, incorporating antibacterial agents into collagen matrices has been explored to enhance therapeutic outcomes, with evidence suggesting improved healing rates when used in DFU treatment.^[^ ^118^ ^]^ For instance, bioinspired bilayer antibacterial collagen scaffolds have been developed to prevent wound infections while providing a biomimetic ECM environment that supports cell recognition, proliferation, and migration, thus promoting angiogenesis and re‐epithelialization in DFUs.^[^ ^119^ ^]^

#### 3.4.3 Polysaccharide‐Based Natural Dressings

Polysaccharide‐based natural dressings are primarily derived from or modified by polysaccharides, offering excellent biocompatibility and biodegradability.^[^ ^120^ ^]^ Commonly used materials include chitosan, alginate, and cellulose.

Chitosan, a cationic polymer, interacts with negatively charged lipids and proteins on bacterial cell walls, while low molecular weight CS may also penetrate the bacterial nucleus, inhibiting RNA and protein synthesis, thereby exerting antimicrobial effects.^[^ ^121^ ^]^ Additionally, CS can gently release N‐acetyl‐β‐d‐glucosamine, which promotes the synthesis of endogenous hyaluronic acid, enhances fibroblast proliferation, and ultimately improves wound healing while preventing scar formation.^[^ ^122^ ^]^

Alginate is a polysaccharide that consists of homopolymeric regions of mannuronic acid and guluronic acid. Alginate‐based dressings form an ordered structure in aqueous environments and, upon absorbing wound exudate, create a firm gel on the wound surface that adheres to the wound without falling off, while being easy and painless to remove.^[^ ^123^ ^]^ Calcium alginate (CA) dressings exhibit both antibacterial and hemostatic properties. The calcium ions within CA can be exchanged with sodium ions in wound exudate, releasing into the wound bed and establishing a moist environment essential for healing.^[^ ^124^ ^]^

**Figure 4:**
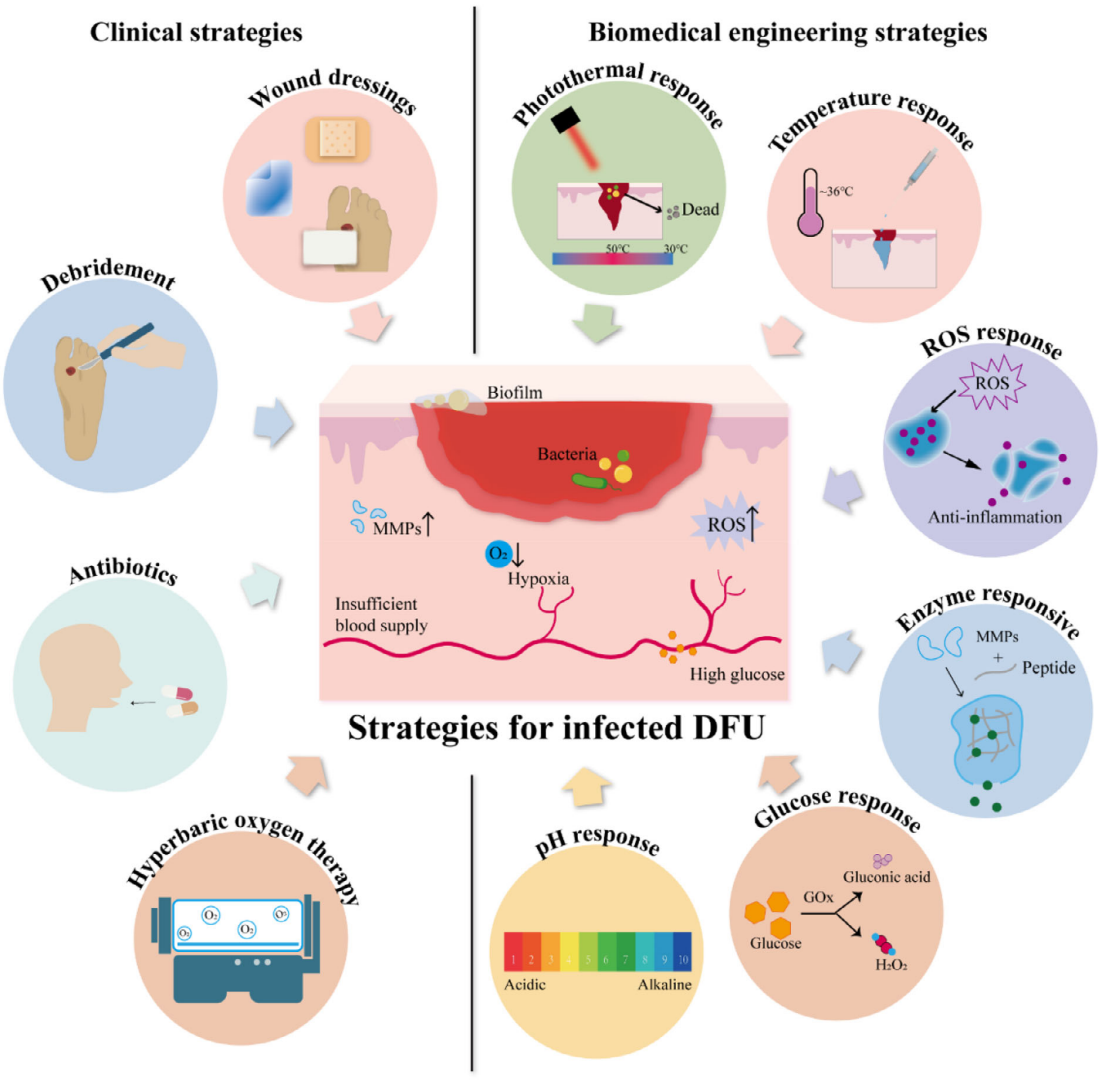
Strategies for infected DFU. ROS, reactive oxygen species; MMPs, matrix metalloproteinases; GOx, glucose oxidase.

Various polysaccharide components can be composited or chemically modified, or combined with other bioactive agents, to enhance multifunctionality and performance (**Figure** **4**).^[^ ^125^ ^]^ These make polysaccharide‐based dressings well‐suited to meet the complex therapeutic demands of infected DFU.

## 4 Responsive Antimicrobial Biomaterials for DFU

### 4.1 Functional Design of Antimicrobial Biomaterials

In the context of DFUs, which are characterized by chronic wound conditions and impaired tissue regeneration, the rational design of multifunctional antimicrobial biomaterials is crucial. These materials should be capable of destroying biofilm formation, improving local ischemia and hypoxia, and regulating immune responses to promote wound healing. By integrating multiple therapeutic functions into a single platform, these biomaterials can exert synergistic effects to modulate the wound microenvironment, ultimately accelerating tissue repair and improving clinical outcomes.

#### 4.1.1 Inhibition and Destruction of Biofilm Formation

In biofilm environments, bacterial communities are protected by an extracellular polymeric substance matrix, which significantly enhances their resistance to antibiotics and immune cell clearance. Functional antimicrobial biomaterials can inhibit and disrupt biofilm formation by locally releasing antimicrobial agents or activating photothermal mechanisms that interfere with bacterial physiology and biofilm architecture.

Certain natural plants and their extracts, including Coptis chinensis, gallic acid, and lutein, exhibit notable antibacterial properties and the ability to disrupt or inhibit biofilm formation.^[^ ^126^, ^127^ ^]^ Sophoraflavanone G and kurarinone, for example, can suppress cell wall biosynthesis, induce bacterial autolysis, and prevent biofilm formation.^[^ ^128^ ^]^ These compounds can also interfere with energy metabolism in MRSA, thereby disrupting the normal physiological activity of bacteria.

**Figure 5:**
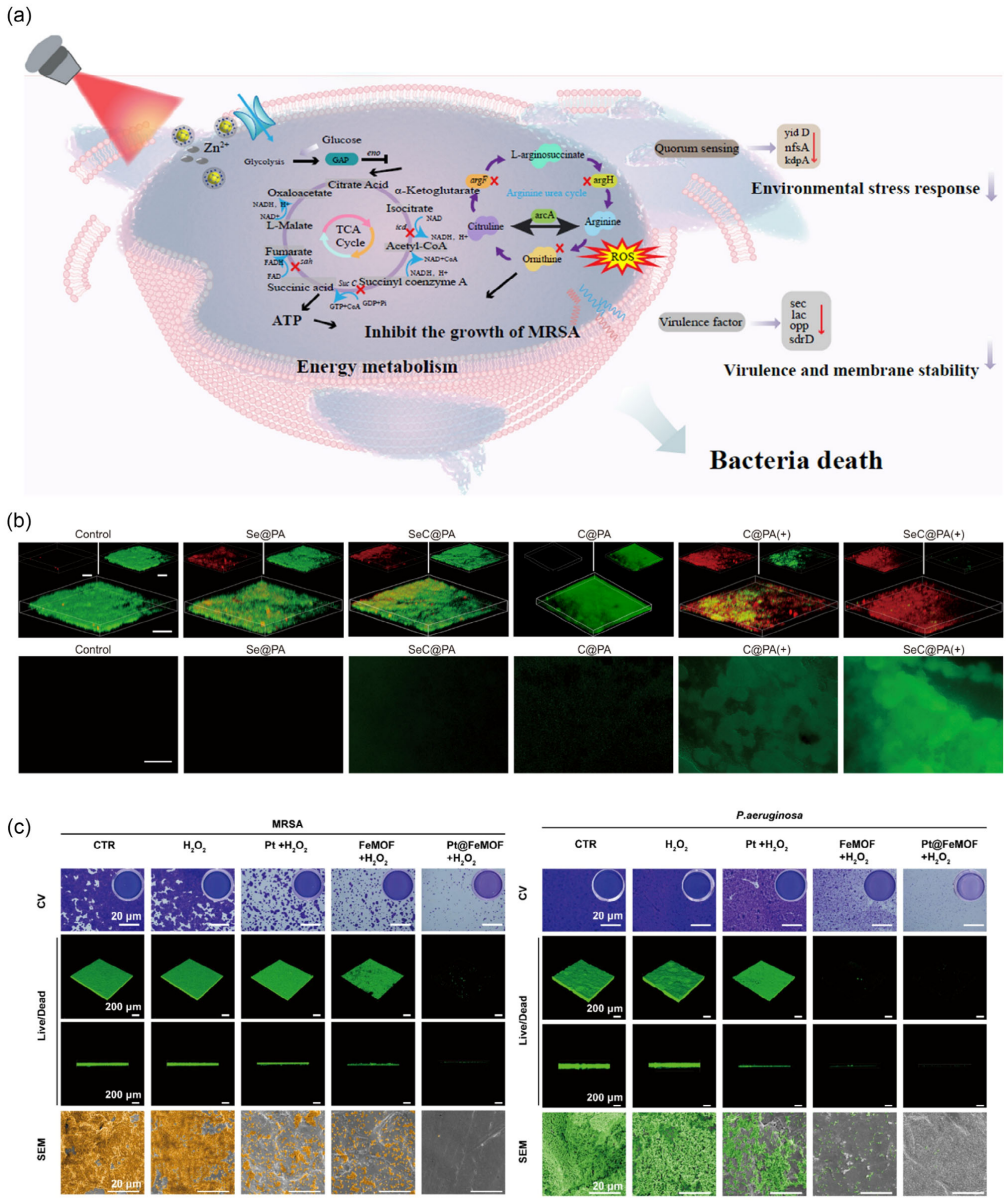
Biomaterials for inhibition and destruction of biofilm. a) Antimicrobial mechanism diagram of COG‐Z@P200 hydrogel. Reproduced with permission.^[^ ^132^ ^]^ Copyright 2025, Springer Nature. b) Anti‐biofilm effect of SeC@PA. Reproduced with permission.^[^ ^129^ ^]^ Copyright 2023, Springer Nature. c) Antibiofilm properties of CTR, H_2_O_2_, Pt + H_2_O_2_, FeMOF + H_2_O_2_, and Pt@FeMOF + H_2_O_2_ groups. Reproduced with permission.^[^ ^131^ ^]^ Copyright 2025, Elsevier.

Depletion of endogenous glutathione (GSH) within the biofilm can enhance the biofilm‐disrupting efficacy of reactive species (RS) such as reactive oxygen species (ROS) and RNS. Hybrid nanoparticles of chlorin e6 (Ce6) with selenium degrade GSH in biofilms through a cascade reaction to produce more lethal RS, thereby eradicating the biofilm (**Figure** **5**b).^[^ ^129^ ^]^ Similarly, dimethyl‐mediated GSH depletion intensifies the damage to extracellular DNA by hydrogen sulfide (H_2_S), which improves ROS activity to disrupt the biofilm structure.^[^ ^130^ ^]^ In another approach, Li et al. developed multifunctional platinum‐iron‐based metal organic framework (Pt@FeMOF) nanocomposites that induce bacterial ferroptosis by combining lipid peroxidation, GSH depletion, iron overload, and arginine metabolism disruption, effectively combating biofilm‐associated infections (Figure 5c).^[^ ^131^ ^]^

In addition, disrupting QS, the chemical communication between bacterial cells, also offers a route to impair biofilm development and bacterial virulence. Under mild photothermal stimulation, hydrogels embedded with polydopamine‐coated ZIF‐8 nanoparticles inhibit metabolic pathways, which include downregulation of glycolysis (eno, gap), TCA cycle blockade (sucC, sdhA, icd), and arginine biosynthesis inhibition (arcA, arcC, arcD).^[^ ^132^ ^]^ This can impair the formation and pathogenicity of biofilm. Concurrent suppression of QS and virulence genes (agr, sec, lac, opp, sdrD) impairs MRSA, while upregulation of stress response genes (yidD, nfsA, kdpA) indicates a state of metabolic paralysis of bacteria (Figure 5a).^[^ ^132^ ^]^ Moreover, hyperbranched poly‐L‐lysine (HBPL) has been shown to downregulate QS systems, inhibit virulence gene expression, and disturb bacterial metabolism, contributing to biofilm disruption.^[^ ^133^ ^]^

Taken together, the design of anti‐biofilm biomaterials should focus on modulating bacterial metabolic activity, disrupting the extracellular polymeric matrix, and disrupting QS to dismantle inter‐bacterial communication. Furthermore, integrating these strategies with physical antimicrobial modalities may enhance efficacy while reducing drug dosage, thereby addressing the challenges of poor antibiotic penetration and bacterial resistance within chronic diabetic wound biofilms.

#### 4.1.2 Improvement of Local Ischemia and Hypoxia

Functional biomaterials contribute to the amelioration of local ischemia and hypoxia by promoting angiogenesis, thereby disrupting the survival niche of bacteria within DFUs.

Bioactive molecules such as epidermal growth factor (EGF), vascular endothelial growth factor (VEGF), and platelet‐derived growth factor (PDGF) play direct roles in wound healing through angiogenesis promotion and re‐epithelialization. Encapsulation within biomaterials protects these growth factors from proteolytic degradation while enabling their localized and controlled release.^[^ ^134^, ^135^ ^]^ Specifically, alginate (Alg)/gum Arabic hydrogels serving as reservoirs for mesoporous silica nanoparticle‐loaded nerve growth factor (NGF) demonstrated enhanced NGF stability and sustained release during wound healing.^[^ ^136^ ^]^ This system significantly upregulated angiogenesis‐related gene expression and promoted cutaneous nerve fiber regeneration.

Therapeutic nucleic acids, including plasmid DNA, small interfering RNA (siRNA), microRNA mimics, messenger RNA (mRNA), and antisense oligonucleotides, can be engineered to enhance the expression of angiogenic factors and thereby address local ischemia.^[^ ^137^ ^]^ However, free nucleic acids are inefficiently internalized by cells, so using appropriate carriers not only enhances cellular uptake by dermal fibroblasts but also protects nucleic acids from degradation.^[^ ^138^ ^]^ Typically, these delivery systems are designed with polycationic domains to facilitate ionic complexation with the negatively charged phosphate backbone of therapeutic nucleic acids and promote endosomal escape post‐internalization.^[^ ^137^ ^]^ Shi et al. utilized biodegradable poly(lactic‐co‐glycolic acid) (PLGA) nanospheres to encapsulate plasmid DNA encoding VEGF‐A and PDGF‐B, achieving enhanced expression of growth factors within the wound beds of DFU.^[^ ^139^ ^]^ This evidence suggests that nanoparticles may serve as promising non‐viral vectors for gene transfer therapies in DFU treatment.

Exosomes derived from macrophages, human umbilical cord mesenchymal stem cells, epidermal stem cells, or other stem cell sources represent promising cell‐free therapeutic tools.^[^ ^140^, ^141^ ^]^ Hydrogel matrices enhance exosome viability and prolong bioactivity in extracellular environments.^[^ ^142^ ^]^ Exosomes derived from polymorphonuclear neutrophils retain the antimicrobial capacity of their parent cells due to the presence of key enzymes and proteins such as myeloperoxidase, elastase, cathepsin G, and lysozyme. ECM‐based hydrogels have been utilized to encapsulate activated neutrophil‐derived exosome mimetics, which effectively suppress bacterial growth in diabetic wound tissues.^[^ ^143^ ^]^ Moreover, these exosomes can serve as delivery vehicles for VEGF, enhancing angiogenesis by improving factor stability and targeting efficiency.

**Figure 6:**
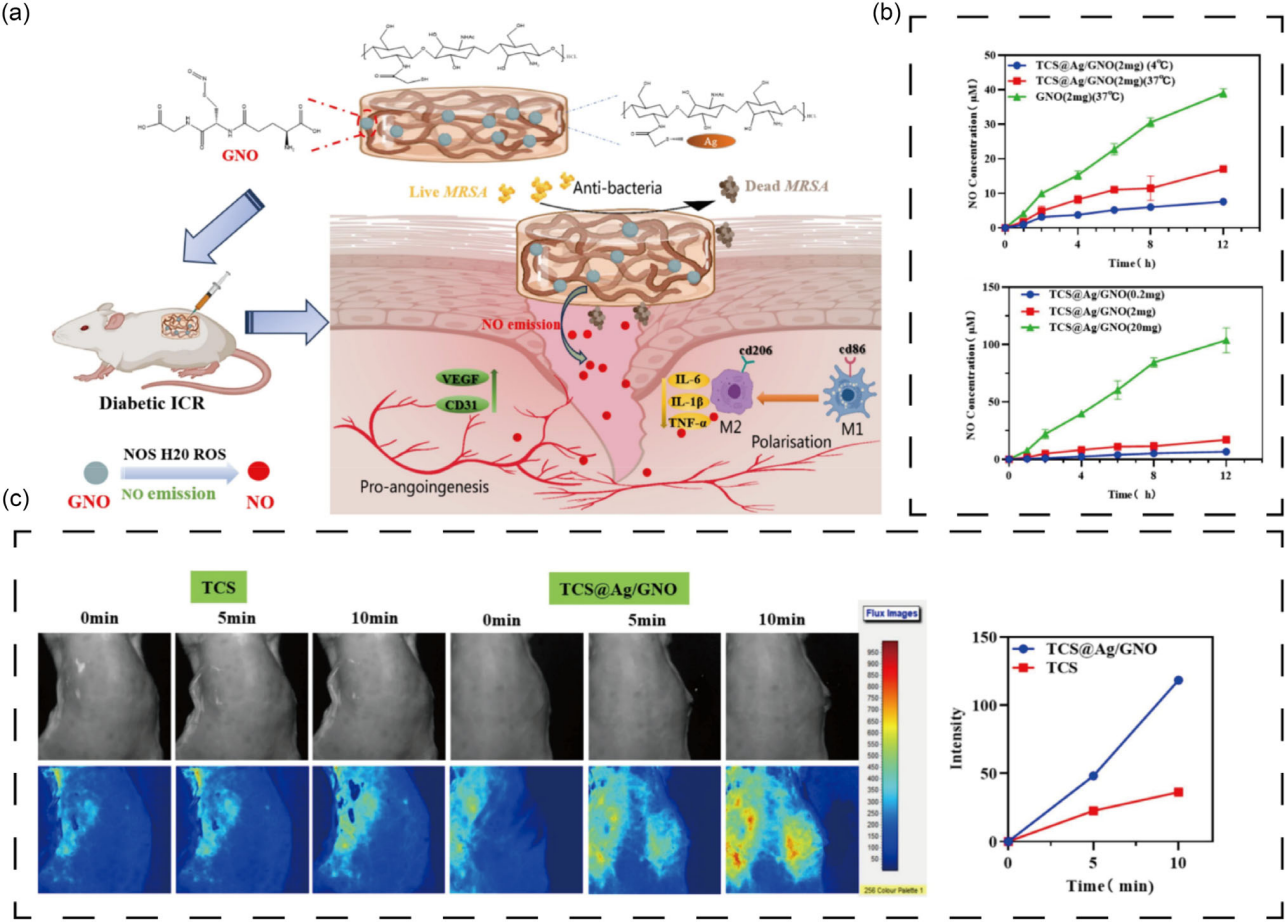
Antibacterial biomaterials for improving local ischemia. Reproduced with permission.^[^ ^145^ ^]^ Copyright 2025, Elsevier. a) Schematic illustration of an injectable chitosan hydrogel combined with NO and AgNPs. b) The cumulative release of NO from hydrogels at different temperatures and contents of GNO in PBS solution. c) The impact on the blood flow of mice by TCS and TCS@Ag/GNO hydrogels at different times.

Gas‐releasing biomaterials represent another innovative approach for alleviating tissue hypoxia and improving blood perfusion in infected DFU. Nitric oxide (NO), for instance, is known to disrupt biofilms by triggering biofilm dispersion or reverting bacteria to planktonic states, making them more susceptible to conventional antimicrobials.^[^ ^144^ ^]^ In addition to its antimicrobial effects, NO also promotes angiogenesis, enhances local blood flow, suppresses inflammatory cytokines, and accelerates wound healing (**Figure** **6**).^[^ ^145^ ^]^ Furthermore, Prussian blue nanoparticles (PB NPs) loaded with Ce6 have been employed in photodynamic therapy to catalyze the decomposition of H_2_O_2_ in the wound microenvironment, resulting in elevated oxygen levels and alleviation of local hypoxia.^[^ ^146^ ^]^

Overall, biomaterials designed to improve ischaemia and hypoxia involve promoting angiogenesis through the delivery of growth factors or enhancing blood circulation. Furthermore, they may incorporate gas‐releasing molecules or oxygen‐producing materials to directly alleviate hypoxia and synergistically combat infection. Adequate perfusion and oxygen supply are prerequisites for fibroblast proliferation, collagen synthesis, and the activity of immune cells. This is crucial for accelerating granulation tissue growth and achieving wound closure.

#### 4.1.3 Long‐Term Antimicrobial Inhibition of Drug Resistance

Monitoring pH levels in the wound bed provides a non‐invasive method for early detection of bacterial infections and offers a continuous means of infection surveillance throughout the wound healing process.^[^ ^147^ ^]^ The pH‐sensitive, color‐changing nature of some substances has been integrated into smart dressings to allow real‐time visual assessment. For instance, Huang et al. designed an injectable smart wound dressing based on PEG (Figure 8b).^[^ ^148^ ^]^ Anthocyanins, extracted from food sources, were employed as visual pH indicators capable of detecting pH variations in the range of 5–9. Simultaneously, poly(L‐lactic acid) microcapsules were integrated as ultrasound‐responsive antibiotic carriers. The wound pH can be visually assessed by analyzing RGB values captured through imaging. Upon detection of infection, ultrasound stimulation can be applied to trigger on‐demand antibiotic release, achieving precise spatiotemporal control over the therapeutic response.

In addition, the long‐term use of conventional antibiotics poses a major risk of inducing multidrug‐resistant bacterial strains and may also result in hepatic and renal toxicity. Consequently, alternative antimicrobial agents such as antimicrobial peptides (AMPs) and metal‐based nanoparticles are being explored as sustainable approaches for infection control in DFU.

AMPs exhibit broad‐spectrum antibacterial activity and represent a promising alternative to traditional antibiotics for infection treatment. AMPs disrupt bacterial cell membrane integrity by altering its conformation, resulting in leakage of intracellular contents and eventual bacterial rupture and death.^[^ ^149^ ^]^ However, their susceptibility to proteolytic degradation necessitates the use of delivery systems to ensure sustained and localized release at the wound site.^[^ ^150^ ^]^ To address this, Guerrero et al. designed a polyanionic hydrogel based on N‐isopropylacrylamide, which electrostatically interacts with positively charged AMPs, enabling controlled and prolonged antimicrobial release.^[^ ^151^ ^]^ By responding to bacterial infection at the wound site, the delivery system enables controlled AMP release, prolongs the antimicrobial activity in serum, and reduces cytotoxicity.^[^ ^152^ ^]^

Metal‐based nanoparticles, such as those composed of gold, silver, and copper oxide, possess excellent antibacterial and pro‐angiogenic properties.^[^ ^153^ ^]^ They can also stimulate the expression of proteins involved in wound healing. Among them, silver nanoparticles (AgNPs) are particularly notable for their broad‐spectrum antimicrobial activity. AgNPs have been shown to disrupt established bacterial biofilms, induce ROS within bacterial cells, and compromise bacterial membrane integrity, ultimately leading to cell lysis.^[^ ^154^ ^]^ Due to these characteristics, AgNP‐containing biomaterials have attracted considerable attention for managing infected DFU.^[^ ^155^ ^]^ For example, incorporating AgNPs into electrospun nanofibers of casein/polyvinyl alcohol (PVA) enhances the antibacterial activity of the dressing while maintaining good cytocompatibility.^[^ ^156^ ^]^ Moreover, a nanocomposite using AgNPs modified with AMPs exhibits superior antibacterial activity compared to either component alone, and it can also disrupt bacterial biofilms by downregulating the expression of key biofilm‐associated genes at the mRNA level.^[^ ^157^ ^]^

Achieving long‐term antimicrobial efficacy while inhibiting resistance lies in circumventing traditional antibiotic resistance pathways. This can be accomplished by employing antimicrobial agents less prone to inducing resistance, such as AMPs and metallic nanoparticles. Such strategies minimize the risk of generating resistant strains, thereby preventing infection recurrence and disease progression caused by antibiotic failure.

#### 4.1.4 Improvement of Wound Healing and Immune Regulation

Functional antibacterial biomaterials can also modulate host immune responses, particularly through regulating macrophage polarization, thereby suppressing excessive inflammation and promoting tissue regeneration. Immunomodulatory strategies generally follow two approaches: delivering immunomodulatory agents to DFU sites or constructing materials from components with inherent immunomodulatory properties.^[^ ^158^, ^159^, ^160^ ^]^

Among natural immunomodulators, β‐glucan (BG) has been extensively studied for its macrophage‐polarizing capacity. BG can induce the M1 to M2 transition in macrophages through down‐regulation of the NF‐κB signaling pathway. Immunomodulatory hydrogels were generated by multivalent ligand interactions of bisphosphonate‐modified BG with Zn^2+^/Mg^2+^.^[^ ^161^ ^]^ Besides, sustained co‐release of Zn^2+^ and Mg^2+^ synergistically promotes proliferation and angiogenesis through up‐regulation of the P13k‐Akt signaling pathway.

**Figure 7:**
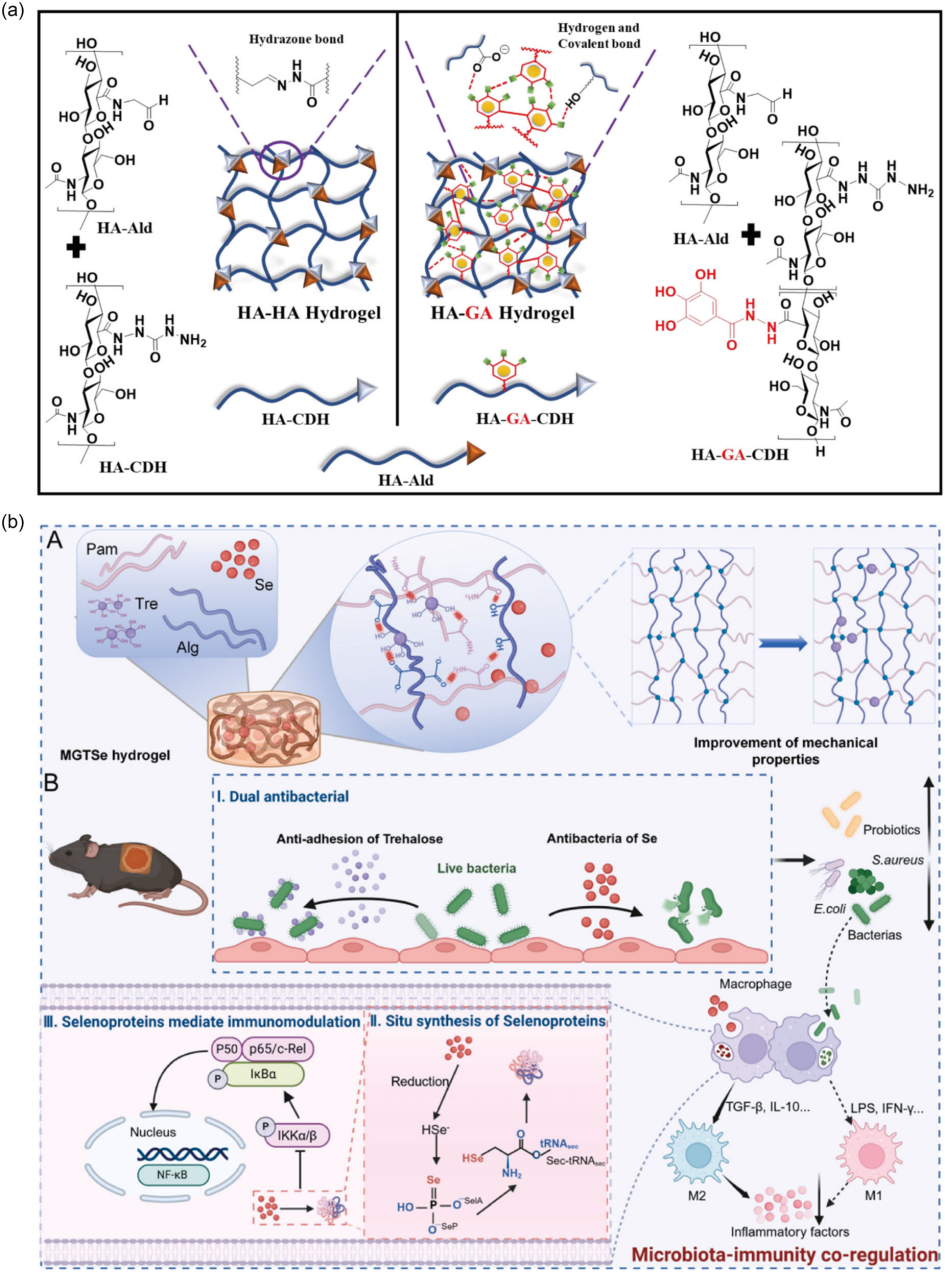
Antibacterial biomaterials for improving immune regulation. a) Schematic representation of the formation of hydrazone cross‐linked HA‐HA and hydrazone and gallol cross‐linked interpenetrating HA‐GA hydrogel. Reproduced with permission.^[^ ^163^ ^]^ Copyright 2022, Elsevier. b) MGTSe hydrogel dressing‐mediated in situ synthesis of selenoproteins for regulating DFU immunity‐microbiota. Reproduced with permission.^[^ ^164^ ^]^ Copyright 2025, Elsevier.

In addition, Hyaluronic acid, a component of the ECM, has emerged as a substance that allows the synthesis of biomaterials with tunable immune functions. The effect on macrophage behavior of hyaluronic acid depends on its molecular weight; high molecular weight hyaluronic acid (≥1250 kDa) at high concentrations (100 μg mL^−1^) has been reported to suppress immune responses.^[^ ^162^ ^]^ Furthermore, functionalization of hyaluronic acid with gallic acid (GA), a polyphenolic immunomodulator, enables the construction of hydrogels that selectively promote anti‐inflammatory macrophage polarization by upregulating IL4ra, IL‐10, TGF‐β, and TGF‐βR1 expression (**Figure** **7**a).^[^ ^163^ ^]^

Additionally, Liu et al. developed a selenium‐based immunomodulatory system by physically encapsulating zero‐valent selenium nanoparticles (SeNPs) within an alginate‐polyacrylamide hydrogel matrix (Figure 7b).^[^ ^164^ ^]^ The released selenium can participate in the biosynthesis of selenoproteins via the SeCys2 pathway, thereby reducing the M1 macrophage phenotype while enhancing M2 polarization. This biomaterial also altered the microbial composition at the wound site by increasing beneficial bacteria such as Lactobacillus and suppressing pathogenic species, including *E. coli* and *S. aureus*. By restoring immune–microbiota homeostasis, it offers a promising biocompatible and non‐toxic strategy for effective DFU treatment.

**Figure 8:**
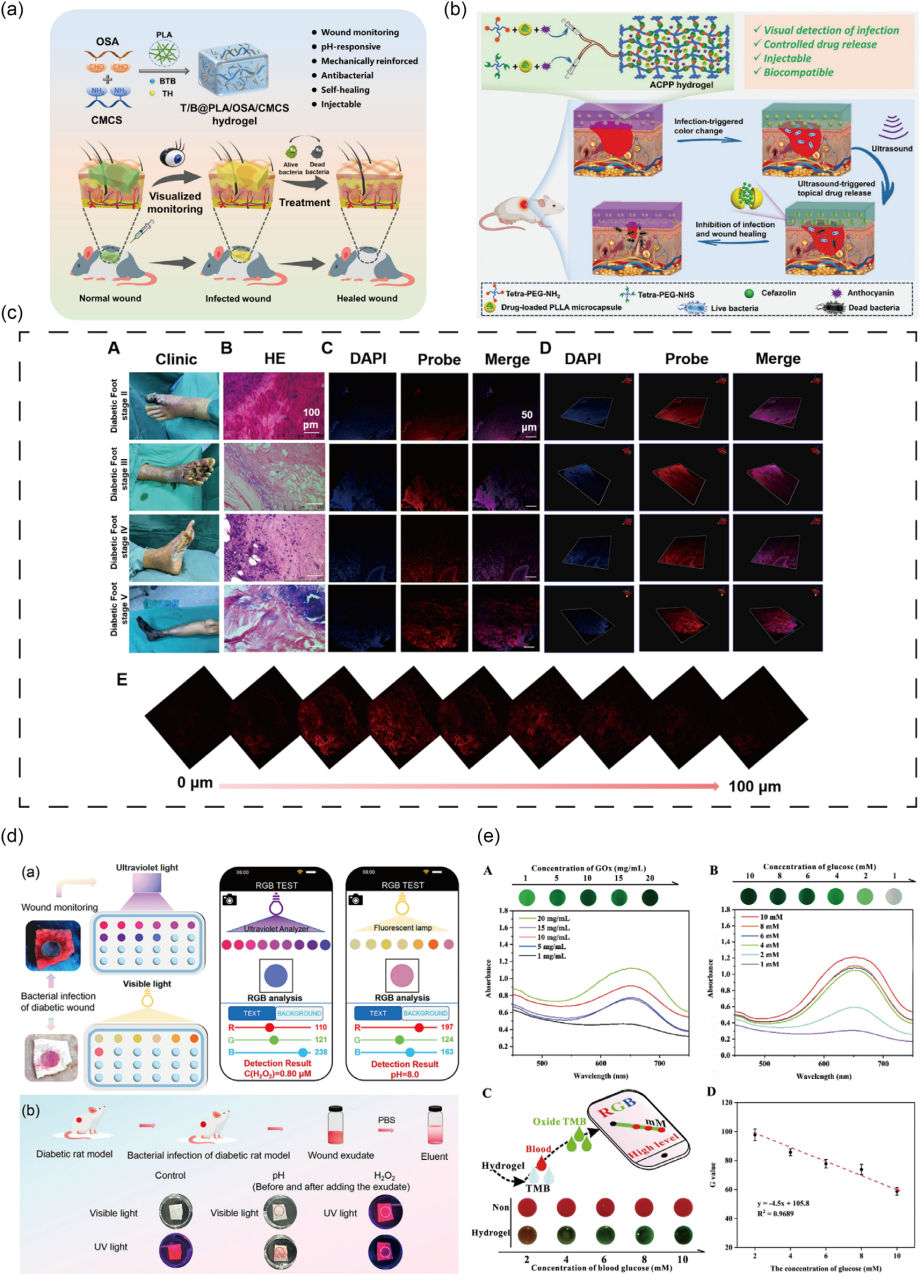
Antimicrobial biomaterials with monitoring functions. a) Illustration of T/B@PLA/OSA/CMCS hydrogel for visual monitoring of the wound microenvironment of bacterial infection and drug‐controlled release therapy. Reproduced with permission.^[^ ^147^ ^]^ Copyright 2024, Elsevier. b) Schematic illustration of real‐time monitoring and on‐demand treatment of skin wounds using ACPP hydrogel. Reproduced with permission.^[^ ^148^ ^]^ Copyright 2024, John Wiley & Sons. c) The probe DCM‐H_2_O_2_ to monitor and image H_2_O_2_ in different diabetic foot stages. Reproduced with permission.^[^ ^165^ ^]^ Copyright 2024, American Chemical Society. d) Janus membrane for visual monitoring of wound status and monitoring H_2_O_2_ and pH levels of wound exudate. Reproduced with permission.^[^ ^166^ ^]^ Copyright 2024, American Chemical Society. e) Detection ability of the TMB/Fe^2+^/PF127/GO_X_ hydrogel for glucose. Reproduced with permission.^[^ ^168^ ^]^ Copyright 2023, American Chemical Society.

Beyond immunomodulation, the real‐time monitoring of wound status is essential for precision therapy. H_2_O_2_ levels fluctuate across different pathological stages of wound healing and thus may serve as a potent biomarker to indicate wound status (Figure 6c).^[^ ^165^ ^]^ To exploit this feature, a multifunctional Janus membrane was developed by incorporating zinc‐based metal‐organic frameworks (Zn‐MOFs), europium‐based MOFs (Eu‐MOFs), and phenol red into a composite structure consisting of a 3D chitosan sponge and polycaprolactone nanofibers (**Figure** **8**d).^[^ ^166^ ^]^ This system enables monitoring of pH (range: 5–8) and H_2_O_2_ levels (0.00–0.80 μM) through the colorimetric response of phenol red and the fluorescence of Eu‐MOFs. Additionally, wound healing progression can be assessed using smartphone analysis of the membrane's RGB signal data.

The rational design of immunomodulatory biomaterials aims to reverse inflammatory stagnation by directing macrophage polarization to M2. Furthermore, active ingredients with immune microenvironment‐modulating functions might be integrated to balance the local microbiota. These systems act as drivers to transition chronic infected wounds from the inflammatory to the proliferative phase, facilitating tissue regeneration.

#### 4.1.5 Glucose Monitoring

Effective glycemic control plays a pivotal role in eliminating infections and promoting ulcer healing. A smart conductive hydrogel patch with both treating and monitoring functions is assembled in situ, forming P(Py‐TA) nanofibrils in the P(AM‐Aa) polymer networks.^[^ ^167^ ^]^ When the P(Py‐TA)/CHA hydrogel was exposed to varying glucose concentrations (20, 50, 100, and 200 mM), a regular decrease in ΔR/R_0_ was observed, demonstrating a strong linear correlation. Notably, the glucose concentrations measured by the hydrogel closely matched those obtained using commercial glucometers, confirming its reliability. This biocompatible hydrogel can adhere directly to the skin and simultaneously provide real‐time sensing of various physiological movements.

In another study, 3,3′,5,5′‐tetramethylbenzidine (TMB) is used as an intermediate bridge to synthesize the hydrogel (Figure 8e).^[^ ^168^ ^]^ Within the glucose concentration range (1–10 mM), TMB oxidizes and causes a color change from colorless to green, which can be monitored visually. The oxidized TMB, activated by glucose, exhibited superior photothermal and chemodynamic antibacterial effects, effectively reducing bacterial burden and accelerating wound healing.

The core of biomaterial design for glucose monitoring lies in harnessing the material's responsiveness to physiologically relevant glucose concentrations as a trigger signal (such as changes in conductivity or color), thereby synchronously activating a therapeutic mechanism. By coupling real‐time glucose monitoring with responsive treatment, these systems enable timely, personalized interventions, thereby improving the management efficiency of DFU.

### 4.2 Responsive Antimicrobial Biomaterials

Intelligent‐responsive biomaterials can respond to changes in biological, physical, or chemical signals at the wound site. Most intelligent‐responsive materials used in DFU treatment are hydrogels, which can modulate behaviors such as self‐degradation to precisely control drug release under specific microenvironmental conditions, thereby enabling dynamic and personalized therapy.^[^ ^169^ ^]^ These systems can respond to endogenous stimuli, such as glucose or enzymes, as well as exogenous stimuli like light or heat.^[^ ^170^ ^]^

#### 4.2.1 Glucose‐Responsive Antimicrobial Biomaterials

The complex microenvironment of DFU is primarily caused by hyperglycemia. Glucose‐responsive materials can undergo phase transitions depending on ambient glucose levels. Three main types have been developed: loaded with glucose oxidase (GOx),^[^ ^171^ ^]^ containing phenylboronic acid (PBA) groups,^[^ ^172^ ^]^ and incorporating glucose‐binding proteins such as Concanavalin A (Con A).^[^ ^173^ ^]^

**Figure 9:**
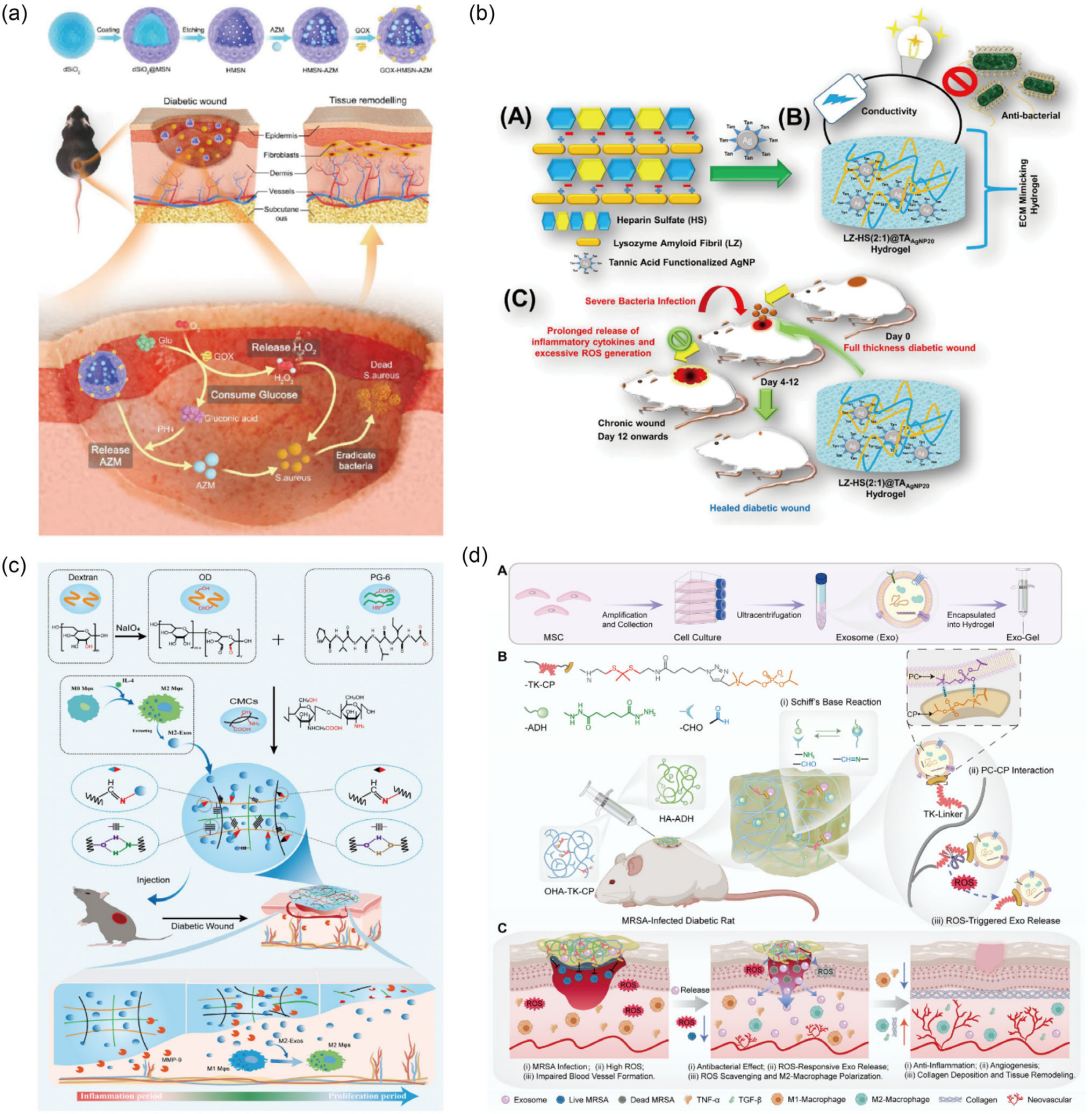
Single‐responsive antimicrobial biomaterials. a) Schematic illustration of the design and application of GOX‐HMSN‐AZM for diabetic wound healing. Reproduced with permission.^[^ ^175^ ^]^ Copyright 2022, Ivyspring. b) LZ‐HS(2:1)@TA_AgNP20_ hydrogel provides a skin ECM‐like conductive cytocompatible environment with pH‐responsive antibacterial property. Reproduced with permission.^[^ ^181^ ^]^ Copyright 2024, American Chemical Society. c) The MMP‐9‐responsive hydrogel (Exo@MRH) with M2‐Exos delivery. Reproduced with permission.^[^ ^184^ ^]^ Copyright 2025, John Wiley & Sons. d) The exosome‐loaded ROS‐responsive hydrogel (Exo‐Gel) system. Reproduced with permission.^[^ ^186^ ^]^ Copyright 2025, American Chemical Society.

GOx exhibits cascade catalytic activity, converting glucose into hydrogen peroxide (H_2_O_2_), thereby lowering local glucose concentrations and generating ROS to kill bacteria.^[^ ^174^ ^]^ Taking advantage of this feature, azithromycin and GOX were encapsulated in hollow mesoporous silica nanoparticles (HMSN), which accelerated diabetic wound healing by modulating the hyperglycemic microenvironment and disrupting bacterial biofilms surrounding the wound (**Figure** **9**a).^[^ ^175^ ^]^ The designed nanosystem demonstrated strong interactions with bacterial cells, disrupting the bacterial cell wall and membrane integrity. This significantly improved the synergistic therapeutic effect of antibiotics and enzymatic reactions.

In addition, Zhou et al. developed a glucose‐responsive nitric oxide (NO)‐releasing hydrogel by in situ crosslinking chitosan conjugated with L‐arginine (L‐Arg) and hyaluronic acid modified with GOx via a Schiff base reaction.^[^ ^176^ ^]^ Under hyperglycemic conditions, this system continuously released H_2_O_2_ and NO through the cascade consumption of glucose and L‐Arg. The combined action of these RS effectively disrupted the membranes of *E. coli* and *S. aureus*, exhibiting potent antibacterial activity. This strategy also contributed to reduced inflammation, enhanced angiogenesis, and increased collagen deposition during the wound healing process.

#### 4.2.2 pH‐Responsive Antimicrobial Biomaterials

pH‐responsive hydrogels typically rely on reversible Schiff base bonds. These bonds dissociate under acidic conditions to loosen the hydrogel network and promote drug release.^[^ ^177^ ^]^ Schiff base reactions occur between aldehyde (–CHO) and amine (–NH_2_) groups. For example, oxidized hyaluronic acid (OHA) provides aldehyde groups that react with amino groups on carboxymethyl chitosan (CMCS) and taurine, forming a rapidly gelling and crosslinked hydrogel.^[^ ^178^ ^]^ This enables the targeted release of taurine in the mildly acidic microenvironment of DFU, enhancing local anti‐inflammatory effects.

However, the pH of infected DFU is typically in the alkaline range and gradually shifts toward acidity as the wound heals.^[^ ^179^ ^]^ Therefore, it is crucial to develop materials capable of dynamically responding to both alkaline and acidic microenvironments to facilitate pH modulation at the wound site.^[^ ^180^ ^]^ Mukherjee et al. developed a multicomponent hybrid hydrogel scaffold by co‐assembling lysozyme‐derived amyloid fibrils (LZ) with heparin sulfate (HS) (Figure 9b).^[^ ^181^ ^]^ Due to the high density of negatively charged groups on HS, its charge state changes in response to variations in pH, which in turn affects its interactions with LZ. These pH‐dependent interactions regulate the structural and functional properties of the hydrogel, ultimately modulating the release rate of antimicrobial agents in a controlled manner.

#### 4.2.3 Enzyme‐Responsive Antimicrobial Biomaterials

Elevated matrix metalloproteinase (MMP) is one of the important factors contributing to the delayed healing of DFU, and is associated with persistent inflammation due to infection.^[^ ^182^ ^]^ Hydrogels were prepared by crosslinking MMP‐2 cleavable peptide‐modified hyaluronic acid and dextran oxide (Dex‐CHO) and loaded with desferrioxamine.^[^ ^183^ ^]^ In the presence of MMP‐2, the hydrogel degrades, releasing deferoxamine to upregulate HIF‐1α expression and promote epidermal regeneration.

Similarly, Meng et al. prepared an intelligent hydrogel by crosslinking oxidized dextran, an MMP‐9‐sensitive peptide (Pro‐Val‐Gly‐Leu‐Ile‐Gly, PG‐6), and CMCS loaded with M2‐Exos (Figure 9c).^[^ ^184^ ^]^ Short peptides of PG‐6 are specifically cleaved at high MMP‐9 concentrations, which causes localized degradation of the hydrogel structure. By recognizing the varying intensity of inflammatory stimuli throughout different stages of wound healing, the hydrogel adaptively modulates drug release profiles.

#### 4.2.4 ROS‐Responsive Antimicrobial Biomaterials

The microenvironment of DFU is often characterized by elevated levels of ROS. Excessive ROS at the wound site can impair essential cell types involved in tissue regeneration, such as fibroblasts and endothelial cells. Moreover, high ROS levels upregulate MMPs, disrupting the balance between ECM synthesis and degradation, reducing collagen deposition, and ultimately hindering wound healing. ROS‐responsive antibacterial biomaterials leverage this pathological feature to achieve targeted delivery of therapeutic agents that aim to eradicate bacteria, modulate inflammatory responses, and regulate angiogenesis.^[^ ^185^ ^]^

Wang et al. developed a multifunctional hydrogel (Exo‐Gel) that utilizes choline phosphate groups to immobilize stem cell‐derived exosomes via electrostatic interactions and achieves antimicrobial effects through physical adsorption and structural disruption of bacterial membranes (Figure 9d).^[^ ^186^ ^]^ In addition, Exo‐Gel with ROS‐responsive thioketal linkers enabled the on‐demand release of exosomes based on local ROS levels, while simultaneously scavenging excessive ROS.

In addition to ROS scavenging, some systems exploit ROS to generate dissolved oxygen, which alleviates hypoxia at the wound site and stimulates neovascularization.^[^ ^187^ ^]^ Furthermore, Degradation of porous silicon loaded with near‐infrared (NIR) reactive dye (IR820) in the presence of ROS was designed to realize the monitoring of ROS levels in wounds by detecting changes in photothermal signals.^[^ ^188^ ^]^

Nevertheless, in the early stages of infection, ROS plays a bactericidal role by inducing oxidative stress through disruption of the bacterial respiratory chain. Therefore, regulating ROS levels at different wound‐healing stages is crucial. To address this, an ultrasound‐modulated nanocomposite “lever‐like” hydrogel was constructed that incorporated ROS‐responsive diselenide liposomes loaded with pro‐regenerative factors, thrombin, and ultrasound sensitizers.^[^ ^189^ ^]^ The hydrogel possesses antibacterial and anti‐inflammatory properties, balances ROS levels at different stages of wound repair, and promotes M2 macrophage polarization via regulation of the PI3K/AKT/NF‐κB pathway to improve DFU healing.

In summary, ROS‐responsive antibacterial biomaterials must not only enable controlled drug release and local ROS scavenging to improve the wound microenvironment, but also preserve the physiological ROS levels necessary for effective antimicrobial action.

#### 4.2.5 Temperature‐Responsive Antimicrobial Biomaterials

Temperature‐responsive antimicrobial biomaterials realize a change in the state of the material through temperature triggering. Thermo‐sensitive hydrogels can undergo a transition from a liquid to a solid state upon exposure to body temperature, which allows them to conform effectively to the complex geometries of wound surfaces.^[^ ^190^ ^]^ Additionally, certain thermos‐responsive materials, such as poly (L‐lactide‐co‐trimethylene carbonate), exhibit thermally driven shape memory behavior. When the temperature rises, they contract centripetally contraction which provides mechanical stimulation to the wound site and promotes wound closure.^[^ ^191^ ^]^

Moreover, precise localization and drug delivery of the material at the wound site are achieved by injectable thermosensitive antimicrobial materials. For example, Cao et al. developed an injectable thermosensitive, ECM‐mimicking hydrogel using hydroxybutyl chitosan, which possessed temperature‐induced phase transition properties, with the incorporation of diatom biosilica to facilitate the transition of the wound microenvironment from the inflammatory phase to the proliferative and remodeling phases.^[^ ^192^ ^]^ Similarly, Zhang and coworkers constructed a thermosensitive hydrogel based on chitosan and ε‐polylysine, which rapidly gelled upon injection into the wound site.^[^ ^193^ ^]^ This hydrogel acted as an efficient carrier for bimetallic nanozymes that enabled simultaneous ROS scavenging, immune modulation, and re‐epithelialization, achieving a wound closure rate of 90.6% within 14 days.

By integrating 3D printing technologies, temperature‐responsive hydrogels can be fabricated into scaffold structures with robust mechanical properties and stable porous architecture.^[^ ^194^ ^]^ These scaffolds are capable of adapting to the curvature of the skin, adhering to fibroblasts, and promoting cell proliferation and migration.

However, the temperature sensitivity of these materials also poses challenges, as they may also respond to external environmental fluctuations, potentially interfering with their stability and performance in vivo.

#### 4.2.6 Photo‐Responsive Antimicrobial Biomaterials

Photothermal therapy (PTT) involves the conversion of light energy into heat by photothermal agents under external irradiation, rapidly generating localized hyperthermia to effectively eradicate bacteria and disrupt biofilms.^[^ ^195^ ^]^ Various nanomaterials, including metallic nanoparticles, carbon‐based nanostructures, black phosphorus, and polydopamine, have been employed for PTT in infected DFU.^[^ ^196^, ^197^, ^198^ ^]^ Beyond antimicrobial action, PTT can also improve local blood circulation, enhance fibroblast proliferation, and promote collagen synthesis, thereby accelerating wound healing.^[^ ^199^ ^]^

Under specific wavelengths of light, photothermal agents can rapidly raise the local temperature to as high as 60 °C or more.^[^ ^200^ ^]^ However, to avoid thermal damage to surrounding healthy tissues, mild PTT (below 50 °C) is preferred at the wound center.^[^ ^201^ ^]^ For instance, a dynamic cross‐linked hyaluronic acid hydrogel dressing (Gel‐HAB) loaded with allomelanin‐N, N′‐dis‐sec‐butyl‐N, N′‐dinitroso‐1, 4‐phenylenediamine nanoparticles (AMNP‐BNN6) achieved nearly 100% bactericidal efficiency at around 48 °C in infected diabetic rat wounds.^[^ ^202^ ^]^ Another novel nanoplatform is designed by co‐decorating 1‐vinyl‐3‐pentylimidazolium bromide and gold nanorods on graphdiyne oxide nanosheets.^[^ ^203^ ^]^ Each cycle was exposed to 808 nm NIR light for 5 min. After ten cycles, the maximum temperature of the nanoplatform was consistently maintained at 50 °C, and it was effective in combating bacterial infections in diabetic wounds and promoting cell proliferation.

**Figure 10:**
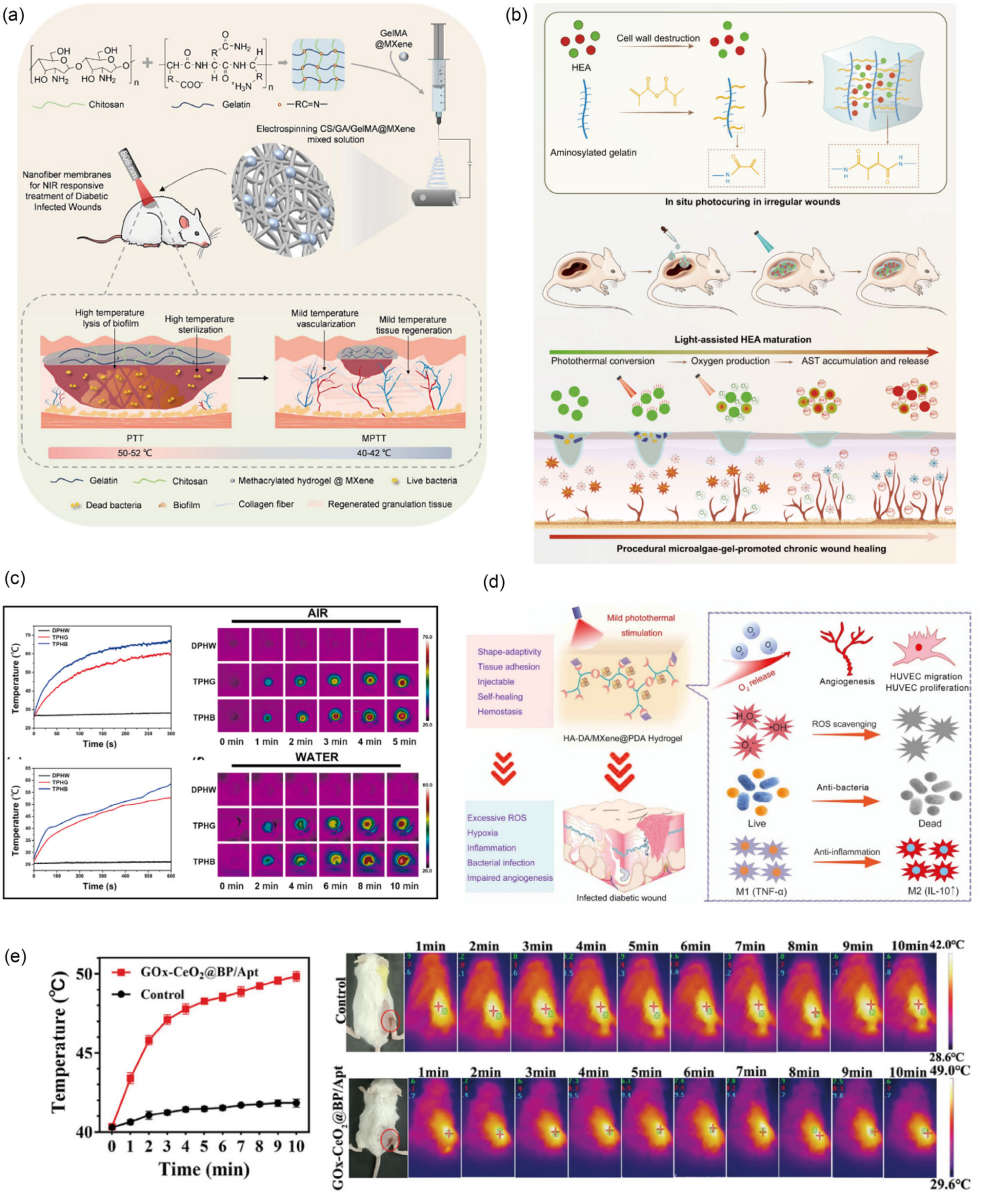
Photo‐responsive antimicrobial biomaterials. a) The MXene microgel‐loaded nanofiber dressing employs temperature‐coordinated photothermal therapy. Reproduced with permission.^[^ ^195^ ^]^ Copyright 2024, Springer Nature. b) HEA@Gel with programmed treatment strategy based on light density. Reproduced with permission.^[^ ^207^ ^]^ Copyright 2024, Springer Nature. c) Photothermal properties of DPHW, TPHG, and TPHB hydrogels. Reproduced with permission.^[^ ^206^ ^]^ Copyright 2025, Elsevier. d) Mechanism of HA‐DA MXene PDA hydrogel. Reproduced with permission.^[^ ^208^ ^]^ Copyright 2022, American Chemical Society. e) In vivo fluorescence imaging and photothermal imaging of infected diabetic mice. Reproduced with permission.^[^ ^198^ ^]^ Copyright 2023, John Wiley & Sons.

Combining PTT with antimicrobial agents can further enhance bactericidal efficacy. In a study by He, AgNPs were loaded onto mesoporous polydopamine (MPDA) particles and embedded in a chitosan‐based hydrogel to improve adhesion.^[^ ^204^ ^]^ The photothermal conversion efficiency of MPDA was 24.0%, which increased to 41.6% after AgNP loading. The combined action of AgNPs and PTT effectively eradicated *E. coli* (99.9%) and *S. aureus* (99.7%), while simultaneously scavenging excess ROS in the cells. Another study has explored photothermal‐triggered release of therapeutic gases such as NO for biofilm disruption, microbial elimination, and promotion of chronic wound healing.^[^ ^205^ ^]^ Notably, certain agents under NIR light are also capable of modulating macrophage polarization, shifting the phenotype from pro‐inflammatory M1 to anti‐inflammatory M2, thereby contributing to an immunomodulatory microenvironment favorable for healing (**Figure** **10**c).^[^ ^206^ ^]^

In addition to antibacterial effects, some photothermal materials can generate oxygen under light irradiation to alleviate hypoxia in DFU. For example, live Haematococcus (HEA) can transform its function: under high light intensity (658 nm, 0.5 W cm^−2^), green HEA (GHEA) produces a photothermal effect and exerts antimicrobial action, decreasing the light intensity (658 nm, 0.1 W cm^−2^), the photosynthetic system of GHEA can continuously produce oxygen (Figure 10b).^[^ ^207^ ^]^ Moreover, Li et al. developed an injectable hydrogel composed of Ti_3_C_2_ MXene nanosheets integrated with dopamine‐modified hyaluronic acid (HA‐DA) (Figure 10d).^[^ ^208^ ^]^ This system employs an oxyhemoglobin/hydrogen peroxide (HbO_2_/H_2_O_2_) reaction, where mild thermal activation from PTT triggers controlled oxygen release, further enhancing the healing process in hypoxic diabetic wounds.

#### 4.2.7 Multi‐Responsive Antimicrobial Biomaterials

DFU presents a particularly challenging microenvironment characterized by chronic hyperglycemia, persistent bacterial colonization, fluctuating pH, and elevated levels of ROS. Thus, multi‐responsive antimicrobial biomaterials combine sensitivity to multiple physiological cues to achieve on‐demand drug delivery based on variable and complex environments.

**Figure 11:**
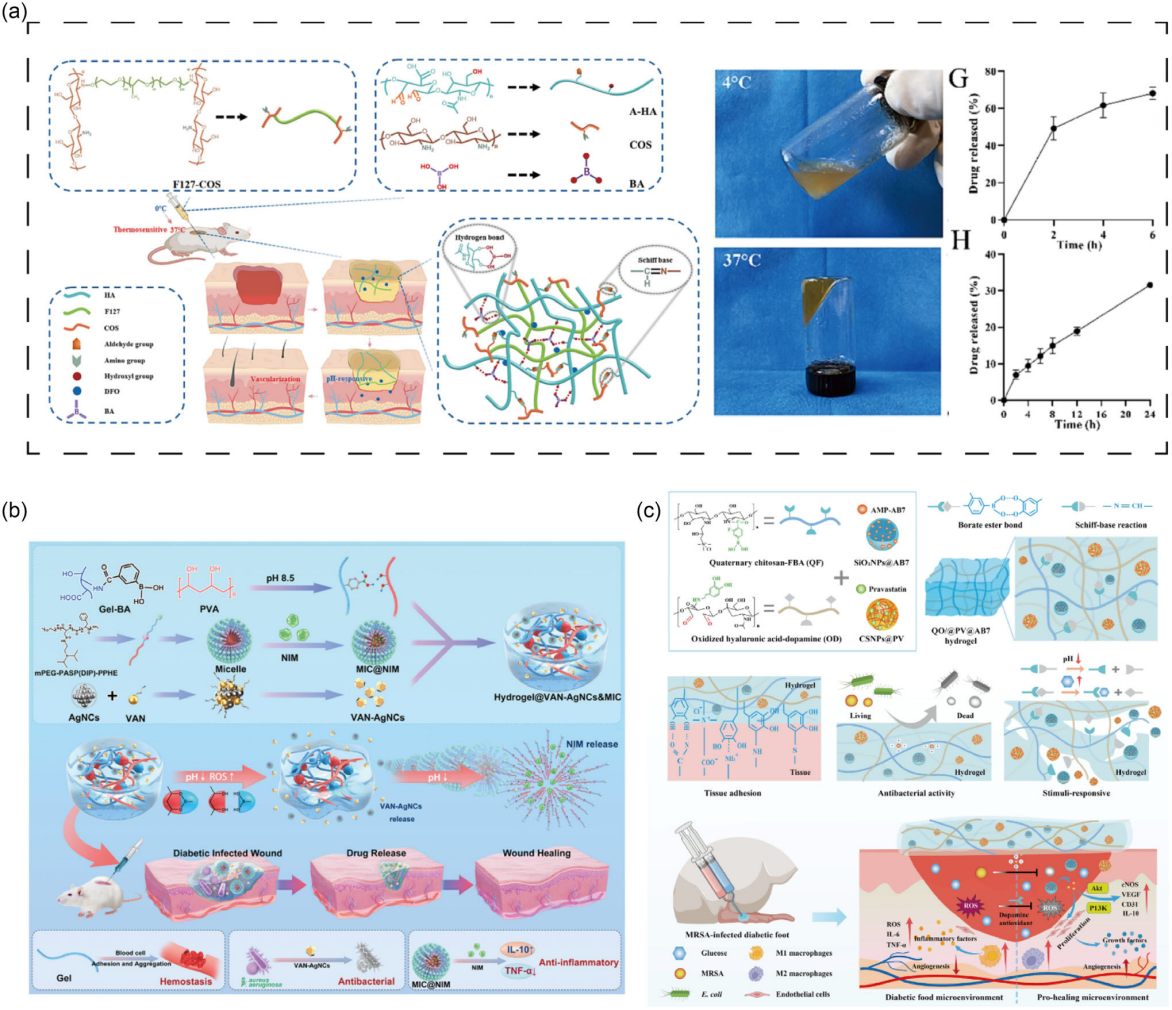
Multi‐responsive antimicrobial hydrogel. a) FCAB/D hydrogel and its medicine release efficiency between pH values = 5.0 (G) and 7.4 (H). Reproduced with permission.^[^ ^209^ ^]^ Copyright 2023, Elsevier. b) Diagrams of pH and ROS dual‐responsive hydrogel containing VAN‐AgNCs‐ and NIM‐loaded micelles. Reproduced with permission.^[^ ^214^ ^]^ Copyright 2021, American Chemical Society. c) Schematic diagram of the glucose and pH dual‐responsive properties of QO/@PV@AB7 hydrogels. Reproduced with permission.^[^ ^192^ ^]^ Copyright 2024, Elsevier.

Firstly, hydrogels that respond to both pH and temperature offer advantages in DFU treatment. These materials remain in sol form at room temperature for easy injection and transition to gel form at body temperature (≈37 °C) for localized drug retention and release. Subsequently, the drug is released as the gel degrades in response to pH changes (**Figure** **11**a).^[^ ^209^ ^]^ A dual‐responsive hydrogel was designed using poloxamer 407 (F127) as a thermo‐sensitive material combined with pH‐sensitive CMCS.^[^ ^210^ ^]^ The hydrogel showed optimal swelling at 37 °C and pH 7.4, facilitating drug diffusion through microchannels. However, its poor adhesion to the wound surface requires additional support.

Furthermore, Guerrero et al. utilized N‐isopropyl acrylamide, which has a lower critical solution temperature of 32–34 °C, to develop a dual‐responsive polyanionic hydrogel incorporating pH‐sensitive carboxylic acid comonomers.^[^ ^151^ ^]^ The hydrogel electrostatically interacts with cationic AMPs, enabling sustained local release. However, activation may require concurrent and significant changes in both pH and temperature.

Glucose/pH dual‐responsive hydrogels can be engineered by crosslinking Schiff bases and phenylboronic acid.^[^ ^211^ ^]^ A hydrogel was designed and prepared by crosslinking PBA‐grafted quaternary chitosan with dopamine‐grafted oxidized hyaluronic acid through phenylboronation, Schiff‐base reaction, and other techniques (Figure 11c).^[^ ^212^ ^]^ Antibacterial properties were further imparted by incorporating silica nanoparticles loaded with the AMP‐AB7. The hydrogel demonstrated a relatively slow rate of weight reduction when exposed to an alkaline environment (pH = 9, no glucose), while degradation significantly accelerated under acidic conditions (pH = 5) or in high‐glucose environments (10–40 mM, pH = 7.4).

Sun et al. designed a microneedle patch loaded with polydopamine‐chelated protoporphyrin IX and gallium, the responsive unit PBA, and insulin.^[^ ^213^ ^]^ This system downregulates the expression of iron uptake regulator and peroxide stress regulator in *S. aureus*, inducing iron starvation and oxidative stress, thereby inhibiting iron‐dependent bacterial activity. By integrating transdermal microneedles with a hydrogel matrix, this intelligent delivery platform enables painless administration while effectively combating drug‐resistant bacteria, showing great potential in modulating host–microbe interactions.

Since DFU is often accompanied by sustained infections and excessive ROS, Wang et al. developed a dual‐responsive hydrogel by grafting 3‐carboxyphenylboronic acid onto the gelatin backbone and crosslinking it with PVA.^[^ ^214^ ^]^ The pH‐ and ROS‐responsive behavior of the hydrogel was attributed to the rapid formation and cleavage of dynamic boronic ester bonds. Moreover, by grafting 2‐(diisopropylamino)ethyl groups (DIP) onto methoxy polyethylene glycol (PEG), an amphiphilic polymer was synthesized. The DIP moieties change hydrophobicity in response to environmental pH fluctuations, functioning as a “pH‐sensitive switch” for controlled drug release. In another study, Wang and coworkers constructed an injectable pH/ROS dual‐responsive glycopeptide hydrogel based on phenylboronic acid‐modified oxidized dextran and caffeic acid‐grafted ε‐polylysine. By spatially encapsulating therapeutics within distinct regions of the hydrogel, a spatiotemporally controlled drug delivery system was achieved.

Furthermore, a composite hydrogel incorporating 3‐carboxyphenylboronic acid‐modified gelatin and PVA was designed to encapsulate vancomycin‐conjugated silver nanoclusters (VAN‐AgNCs) and nimesulide (NIM) (Figure 11b).^[^ ^214^ ^]^ Under inflammatory acidic and high ROS conditions, the hydrogel rapidly degraded to release VAN‐AgNCs for effective antibacterial activity. Subsequently, the acidic environment triggered micelle disintegration and NIM release, which suppressed M1 macrophage activity and improved wound healing. This sequential and stimuli‐responsive drug delivery system provides multifunctional therapeutic actions, including hemostasis, anti‐inflammation, antibacterial effects, and angiogenesis promotion, thus accelerating the healing process of infected diabetic wounds.

**Figure 12:**
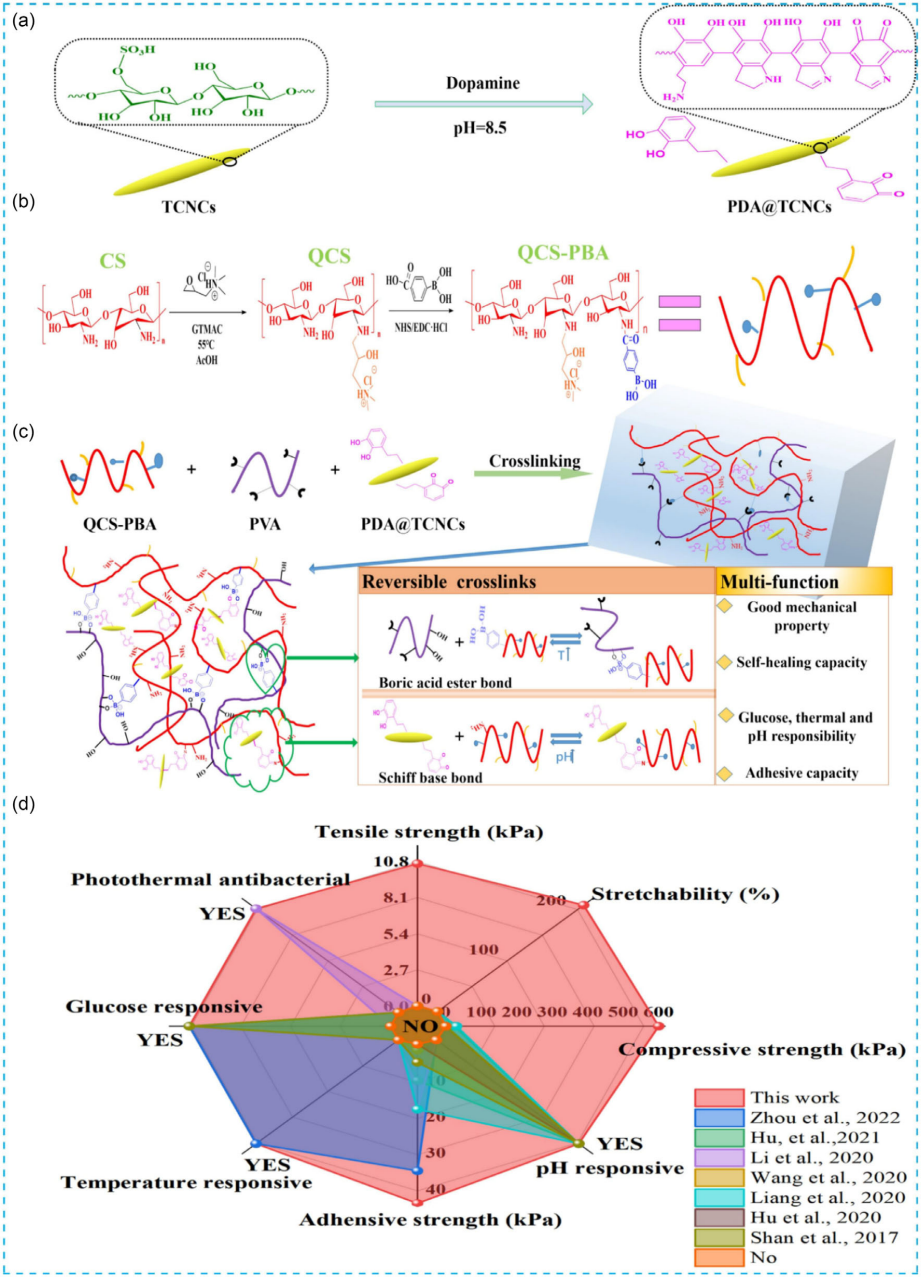
Multi‐responsive (pH, temperature, glucose) antimicrobial hydrogel (QPT_x_). a) Synthesis route of PDAn@TCNCs. b) Synthesis route of QCS‐PBA. c) Synthesis route and cross‐linking mechanism of hydrogel. d) Summary of mechanical properties and adhesive performances of injectable hydrogels.^[^ ^215^ ^]^ Reproduced with permission. Copyright 2024, Elsevier.

Since the wound microenvironment evolves, multi‐responsive hydrogels can better adapt to its complexity and offer dynamic, precise drug release tailored to different healing stages of DFU. Hua et al. developed an injectable multifunctional hydrogel QPTx by combining pH‐, temperature‐, and glucose‐responsive elements using dynamic covalent cross‐linking of Schiff base and boronate ester bonds (**Figure** **12**).^[^ ^215^ ^]^ The system incorporated phenylboronic‐modified quaternized chitosan (QCS‐PBA), polydopamine‐coated TCNCs (PDAn@TCNCs), and PVA. Under low pH (pH 6.0), elevated glucose, and high temperatures, insulin release was significantly enhanced. Additionally, the hydrogel exhibited photothermal antibacterial activity and ROS scavenging capability.

### 4.3 Bionic Materials

Biomaterials can be engineered to mimic the native skin environment, aiming to facilitate wound healing. Among them, materials derived from natural ECM components, such as glycosaminoglycans, have attracted considerable interest due to their unique biological properties.^[^ ^216^ ^]^

**Figure 13:**
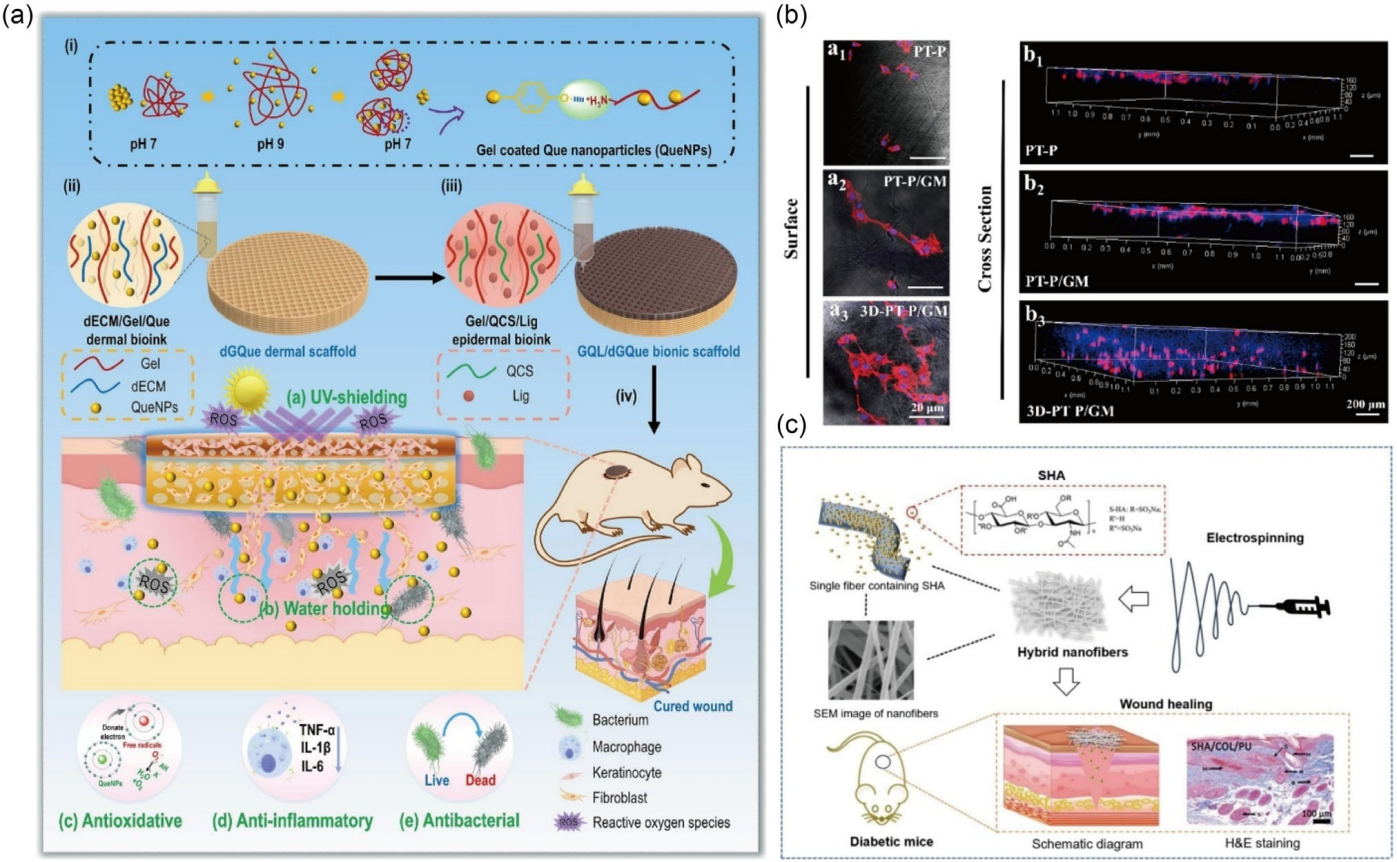
Bionic materials. a) Schematic illustration for the fabrication and mechanism of GQL/dGQue bionic hybrid scaffold. Reproduced with permission.^[^ ^217^ ^]^ Copyright 2025, Elsevier. b) Effect of the 3D hydrogel/nanofiber composite scaffolds on cell behavior in vitro. Reproduced with permission.^[^ ^220^ ^]^ Copyright 2022, Europe PMC. c) SHA/collagen‐based nanofibrous biomimetic skins. Reproduced with permission.^[^ ^221^ ^]^ Copyright 2024, Elsevier.

To replicate the complex structure of human skin, a recent study employed 3D bioprinting technology to fabricate a bilayered, multifunctional bionic skin scaffold, in which the epidermal layer was composed of gelatin, quaternized chitosan, and lignin, while the dermal layer incorporated skin‐derived decellularized ECM, gelatin, and quercetin (**Figure** **13**a).^[^ ^217^ ^]^ This design not only provided effective ultraviolet (UV) shielding but also offered long‐term moisturization and supported cell infiltration and tissue regeneration. Notably, this bionic scaffold exhibited significant antibacterial efficacy, with inhibition rates of 90.76 ± 4.94% against *E. coli* and 90.34 ± 4.14% against *S. aureus*.

Nanofibers, which structurally and compositionally resemble the native ECM, support essential cellular processes including adhesion, proliferation, and migration.^[^ ^218^ ^]^ Zhang et al. fabricated PCL electrospun fibers incorporated with AgNPs to mimic the topology of the ECM while offering mechanical strength and antibacterial properties.^[^ ^219^ ^]^ To further enhance hydrophilicity, the fibers were coated with multiple layers of GelMA. This nanofibrous membrane effectively suppressed AGE‐induced M1 macrophage polarization and promoted the transition toward the anti‐inflammatory M2 phenotype. Moreover, the ordered 3D architecture of the nanofibrous scaffold facilitated uniform cellular distribution, promoting angiogenesis and collagen deposition in diabetic wounds (Figure 13b).^[^ ^220^ ^]^

In addition, nanofiber membranes can serve as artificial skin substitutes to reduce abnormal scar formation and improve the quality of wound healing. Meanwhile, these biomimetic membranes also provide barrier protection, helping to prevent bacterial infection. Zhou and colleagues synthesized sulfated hyaluronic acid (SHA) via chemical sulfation and combined it with a collagen (COL) matrix to fabricate SHA/COL hybrid nanofiber skins (Figure 13c).^[^ ^221^ ^]^ To compensate for the mechanical limitations of SHA/COL nanofibers, polyurethane was introduced. Bionic skin has a stimulatory effect on cellular behavior, including high proliferation rates and maintenance of normal phenotypes of specific cells. It is used as a means of accelerating tissue regeneration and skin re‐epithelialization.

**Figure 14:**
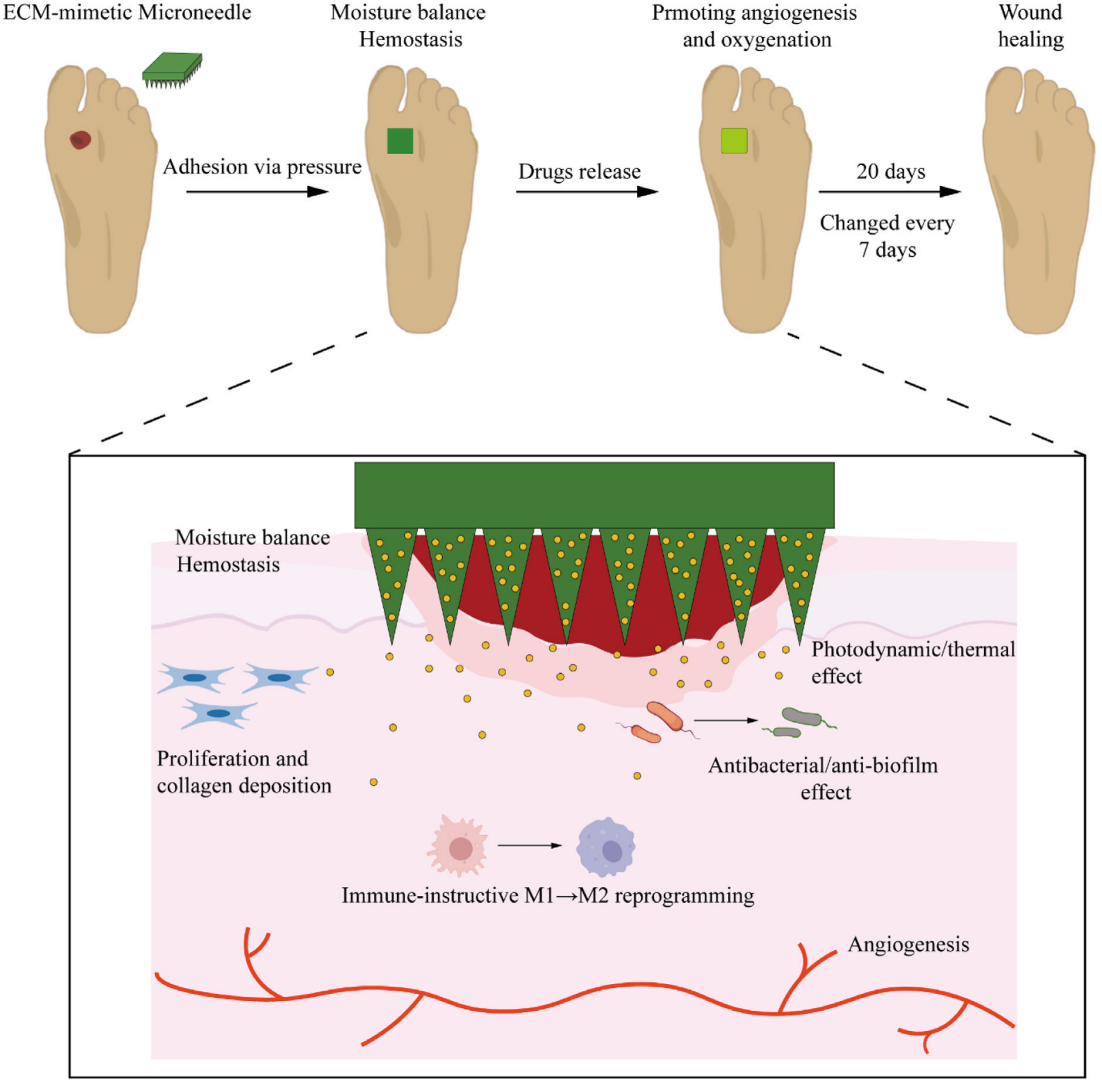
Schematic illustration of ECM‐mimetic microneedle for DFU therapy.

In summary, these multifunctional biomimetic materials transcend the passive role of traditional dressings by actively guiding and accelerating high‐quality tissue regeneration. Classification of the above‐mentioned responsive biomaterial for DFU is summarized in **Table** **2**. This approach involves the strategic design of materials to recapitulate key aspects of the native wound healing cascade, including pro‐regenerative ECM‐mimetic scaffolds, immunomodulatory cues, pro‐angiogenic and oxygenating agents, and antimicrobial/anti‐biofilm strategies (**Figure** **14**). By converging these multifaceted capabilities into a single, smart platform, these biomimetic systems can dynamically interact with the wound microenvironment.

**Table 2:** Classification of responsive biomaterial for DFU.

Stimulus	Material	Payload	Product	Model	Time to Closure	Efficacy outcomes	Ref.
pH	Amyloid fibril and heparin sulfate	Tannic acid functionalized silver nanoparticles	LZ‐HS(2:1)@TA_AgNP20_ hydrogel	Diabetic rats with multispecies bacteria‐infected wounds	18 days	(1) Antibacterial and antibiofilm; (2) Reduced IL‐6 and TNF‐α; (3) Promoted angiogenesis.	[181]
Enzyme	Hyaluronic acid modified with maleimide and oxidized dextran	MMP‐cleavable peptides and deferoxamine (DFO)	MMP‐2‐responsive Hydrogel	Diabetic rats	14 days	(1) Accelerated wound epithelialization and angiogenesis; (2) Enhanced HIF‐1α expression; (3) Reduced inflammatory cell infiltration.	[183]
	Oxidized dextran and carboxymethyl chitosan	MMP‐9‐sensitive peptide Pro‐Val‐Gly‐Leu‐Iso‐Gly and M2 macrophage‐derived exosomes (Exo)	MMP‐9‐responsive hydrogel (Exo@MRH)	Diabetic mice	21 days	(1) Induced macrophage polarization from M1 to M2 phenotype; (2) Downregulated inflammation‐related pathways	[184]
ROS	Silk fibroin methacrylated, modified collagen type III, and lipid nanoparticles	AMP and puerarin	SFMA/rColMA/LNP@AMP@PUE hydrogel	Diabetic rats with infected wounds	≈12 days	(1) Highly effective against *E. coli* and *S. aureus*; (2) Modulated inflammation	[185]
	Hyaluronic acid‐adipic dihydrazide and choline phosphate	Umbilical cord mesenchymal stem cell‐derived Exo	Exo‐hydrogel	Diabetic rats with MRSA‐infected wounds	≈90% wound closure within 21 d.	(1) MRSA colonies were suppressed; (2) Promoted macrophage polarization toward M2 type; (3) Scavenged ROS.	[186]
Temperature	Electrospun Poly‐(lactic acid‐co‐trimethylene carbonate) nanofibers and GelMa	Epinecidin‐1@chitosan (Epi‐1@CS) nanoparticles	Self‐contracting nanofiber/hydrogel (TSNH) composite dressing	Diabetic mice with infected wounds	≈14 days	(1) Improved wound closure rates; (2) Antimicrobial activity, including MRSA and *S. aureus*.	[191]
	Chitosan (CS) and ε‐polylysine (PL)	Copper‐magnesium metal–organic frameworks	Cu/Mg‐MOF@CS/PL hydrogel	Diabetic mice	21 days	(1) Promoted epithelial regeneration and angiogenesis; (2) Improved skin tissue structure.	[193]
Light	GelMA	MXene	NIR‐stimulated microgels	Diabetic rats with infected wounds	14 days	(1) Sterilized by heating to 52 °C in 5 min and destroyed mature biofilms within 10 min; (2) Reduced scar width.	[195]
	GelMA	Haematococcus	658 nm laser‐stimulated HEA@Gel hydrogel	Diabetic mice with infected wounds	≈95% wound closure within 9 d	(1) Re‐epithelialization and dermal tissue regeneration; (2) Improved oxygen supply; (3) Enhanced macrophage polarization toward the M2 type.	[207]

## 5 Discussion

Researchers and clinicians first face severe barriers of scalability and standardization in the transformation of infectious diabetic foot biomaterials. From the material side, the screening of raw materials for popular carriers such as hydrogels and nanofibers has the problem of disjunction between laboratory advantages and clinical accessibility—some synthetic polymers with excellent antibacterial properties show ideal degradability and biocompatibility at the laboratory level, but there are many problems in industrial production, such as difficulty in raw material purification, high cost, and significant fluctuations in purity between batches, which are difficult to meet the economic and stability needs of large‐scale clinical applications. Moreover, considering the sterilization and quality control, infected DFU biomaterials need to give consideration to both antibacterial activity retention and sterility assurance. However, conventional sterilization methods are prone to damaging the cross‐linking structure of hydrogels and lead to agglomeration of nanofibers. Although low‐temperature plasma or radiation sterilization can reduce material damage, there are problems such as high equipment investment, low sterilization efficiency (especially for micro pores of microneedle arrays), and some antibacterial ingredients (such as loaded drugs) are easy to be degraded. In addition, the clinical quality control requirements for biomaterials are far higher than the laboratory standards, such as the swelling rate of hydrogels, the diameter uniformity of nanofibers, the mechanical strength of microneedles, and other key indicators. However, most research teams currently lack quality control schemes that meet GMP standards, which makes it difficult for laboratory results to pass the preclinical compliance verification, creating a dilemma where research is feasible but translation is impossible.

From a clinical application perspective, the lack of biosafety risks and persistent therapeutic effects poses another obstacle to the conversion of DFU biomaterials. On the one hand, DFU wounds require long‐term local exposure to biomaterials (usually requiring continuous application for 7–14 days or even longer), while the current biosafety assessment of most carrier systems only stays at the level of short‐term cytotoxicity or acute animal experiments, ignoring the potential risks under chronic exposure: some drug‐loaded nanofibers release small molecule monomers during degradation, which may trigger chronic inflammatory reactions in local tissues. Because the microneedle array needs to penetrate the cuticle of the skin, its metal or polymer base may activate local immune cells and induce immunogenic reactions when it contacts the ulcer wound for a long time, especially for the immune dysfunction prevalent in diabetes patients. In addition, for infected DFU wounds dominated by biofilms, existing biomaterials generally face the bottleneck of short‐term effectiveness and long‐term drug resistance. Although most antibacterial materials can inhibit planktonic bacteria in the early stage, their ability to penetrate mature biofilms is limited, and the sustained release of antibacterial components can easily induce bacterial resistance gene mutations; At the same time, clinicians need to frequently evaluate the wound healing status to adjust the treatment plan, but the existing materials lack a clear correlation standard of efficacy monitoring dressing change time. Some hydrogels are easy to degrade in advance under the immersion of wound exudate, leading to premature loss of antibacterial ingredients. It is necessary to shorten the dressing change interval, which may greatly limit its wide application in outpatient scenarios.

Although the transformation of infectious diabetes foot biomaterials is full of practical obstacles such as scalability, safety, and efficacy durability, its research still carries irreplaceable scientific value, and its core is to provide an innovative direction to break through the limitations of traditional treatment for this high disability and high‐burden disease. There are significant shortcomings in the current clinical treatment methods for infected DFU: conventional antibacterial dressings can only achieve surface sterilization and cannot penetrate biofilms or maintain long‐lasting antibacterial concentrations on the wound surface; Although surgical debridement can remove necrotic tissue and infected lesions, it is prone to causing normal tissue damage and difficult to solve the problem of persistent wound infection and slow healing after surgery; The use of systemic antibiotics faces three risks: insufficient local drug concentration, systemic toxic side effects, and the growth of drug‐resistant bacteria. Through functional integration design, new biomaterials are expected to achieve synergistic effects of antibacterial, healing, and microenvironment regulation. These design concepts break through the limitations of traditional single‐function treatment and passive intervention, providing a precise and proactive new paradigm for DFU treatment. Their scientific value is not only reflected in technological innovation but also in promoting interdisciplinary integration, thus providing important research support for the treatment of complex chronic infected DFU wounds.

## 6 Conclusion

DFU represents a severe complication of diabetes, impairing patients’ quality of life. Infected DFUs are particularly challenging to manage, often leading to delayed wound healing, osteomyelitis, toe or limb amputation, or even systemic infections and death. The primary factors contributing to infected DFU include peripheral neuropathy, vascular impairment, and immune dysfunction. Moreover, alterations in the local wound microenvironment, the presence of bacterial biofilms, and the emergence of antimicrobial resistance collectively contribute to persistent infections and delayed healing. Current strategies face major challenges, including prolonged treatment courses, rising antibiotic resistance, and frequent recurrences. Therefore, there is an urgent need for more advanced therapeutic approaches to promote efficient wound healing.

Antimicrobial biomaterials have emerged as promising wound dressings due to their excellent biocompatibility, moisture retention, and mechanical properties. By integrating multifunctional designs, these materials can disrupt biofilm formation, alleviate local ischemia and hypoxia, combat antimicrobial resistance, modulate immune responses, and even monitor physiological parameters such as glucose levels. These synergistic mechanisms collectively optimize the wound microenvironment and accelerate tissue repair in infected DFU. Furthermore, the incorporation of stimulus‐responsive elements enables spatiotemporally controlled drug release in response to dynamic changes in the DFU microenvironment, thereby enhancing therapeutic precision. In addition, biomimetic antimicrobial materials, which replicate the structural and functional features of native skin tissue, provide a favorable scaffold for cell proliferation, tissue regeneration, and re‐epithelialization.

The rational design of multifunctional biomaterials offers promising solutions for the management of infected diabetic foot ulcers. A promising near‐term strategy lies in integrated platforms that combine anti‐biofilm activity, immunomodulation, and angiogenesis, thereby simultaneously addressing the three core challenges of infection, persistent inflammation, and ischemia‐hypoxia. However, several critical barriers remain before clinical translation. First, the long‐term biosafety of some nanoparticles and AMPs under chronic use remains insufficiently characterized, especially with respect to systemic toxicity and immune tolerance. Second, the scalable and sterile manufacture of complex biomaterials poses practical hurdles, requiring good manufacturing practice‐compatible processes. Finally, the lack of standardized large‐animal models that faithfully reproduce both infection and ischemia in DFU limits robust preclinical benchmarking. Future efforts should prioritize translational research. Feasible trial designs should incorporate primary endpoints such as wound closure rates and infection recurrence rates, while emphasizing patient‐centered outcomes like pain levels during dressing changes, dressing replacement frequency and associated burden, and overall quality of life. By aligning material innovation with translational feasibility and patient needs, multifunctional antimicrobial biomaterials may transition from experimental concepts to effective clinical therapies.

## Conflict of Interest

The authors declare no conflict of interest.
